# Towards a feminist philosophy of engagements in health-related research

**DOI:** 10.12688/wellcomeopenres.16535.1

**Published:** 2021-03-15

**Authors:** Sonja Erikainen, Ellen Stewart, Sarah Chan, Sarah Cunningham-Burley, Sophie Ilson, Gabrielle King, Carol Porteous, Stephanie Sinclair

**Affiliations:** 1 Centre for Biomedicine, Self and Society, Usher Institute, University of Edinburgh, Edinburgh, EH8 9AG, UK

**Keywords:** engagement, involvement, health research, feminist, epistemology, evaluation

## Abstract

Engagement with publics, patients, and stakeholders is an important part of the health research environment in the UK and beyond today, and different ‘engaged’ health research modalities have proliferated in recent years. Yet, the conceptual landscape currently surrounding engagement is contested. There is no consensus on what, exactly, ‘engaging’ means, what it should look like, and what the aims, justifications, or motivations for it should be. In this paper, we set out what we see as important, outstanding challenges around the practice and theory of engaging and consider the tensions and possibilities that the diverse landscape of engaging evokes. We examine the roots, present modalities and institutional frameworks that have been erected around engaging, including how they shape and delimit how engagements are framed, enacted, and justified. We inspect the related issue of knowledge production within and through engagements, addressing whether engagements can, or should, be framed as knowledge producing activities. We then unpack the question of how engagements are or could be valued and evaluated, emphasising the plural ways in which ‘value’ can be conceptualised and generated. We conclude by calling for a philosophy of engagements that can capture the diversity of related practices, concepts and justifications around engagements, and account for the plurality of knowledges and kinds of value that engagements engender, while remaining flexible and attentive to the structural conditions under which engagements occur. Such philosophy should be a feminist one, informed by feminist epistemological and methodological approaches to equitable modes of research participation, knowledge production, and valuing. This will enable a synergy of empirical, epistemic, and normative considerations in developing accounts of engaging in both theory and praxis. Modestly, here, we hope to carve out the starting points for this work.

## Disclaimer

The views expressed in the article are those of the author(s). Publication in Wellcome Open Research does not imply endorsement by Wellcome.

From patient involvement panels to twitter chats to citizens’ juries, public engagement is part of the health research environment in the UK today. The terms ‘engagement’ and ‘involvement’ are part of a spacious conceptual landscape where multiple terms are used simultaneously and in overlapping ways across academic, policy and public discourses. These include ‘public,’ ‘patient,’ ‘community,’ or ‘stakeholder’ ‘participation,’ knowledge ‘communication,’ ‘exchange,’ and ‘co-production.’ These terms are also connected with phenomena such as ‘citizen science’ and ‘lifelong learning.’ While the different concepts collate diverse, sometimes conflicting practices, mechanisms and frameworks for conceptualising and enacting the relationship between science and wider society, it is no longer considered acceptable for the practice of health research to be ‘un’-engaged.

There is no consensus on what, exactly, ‘engaging’ means, or what it should look like, and this gives rise to a need for critical interrogation of the concepts and practices around engaging (c.f. Crowe *et al.*, 2020; Stilgoe *et al.*, 2014). Different terms frame the people who are to be engaged with, and their roles, in different ways that performatively shape practices of engaging, including, for example, whether they are ‘involved’ in, ‘engaged’ with, or ‘co-producers’ of research. While many terms centre the ‘public’ as those who are engaged with, others focus on specific groups such as ‘patients’ or ‘stakeholders.’ There has also been a shift away from the notion of a singular, undifferentiated public with a unified perspective, towards a recognition of publics as plural, acknowledging the diversity of groups and individuals who are not framed only in contrast to health professionals/researchers (c.f. Felt & Fochler, 2010). Concurrently, despite the multiplicity of terms used to describe engaging and engagement, those who are doing the engaging are rarely explicitly specified. Rather, their identities remain implicit yet presumed, i.e. scientists, health professionals, researchers. Yet a distinction is generally drawn between the ‘engagers’ and the ‘engagees’ as the two sides in the science/society relationship. The central role of public engagement professionals, who often plan and undertake the engaging, is also frequently left silent across all these conceptualisations.

There is no consensus on what the aims, justifications, or motivations of engaging are, or should be. Notably, Fiorino (1990) distinguished between substantive rationales, where engagements are being justified because they lead to better ends through dialogue; instrumental rationales, where engagements are undertaken to achieve some other pre-defined end, such as ‘better trust in science’; and normative rationales, where engagements are justified because they are the ‘right thing’ to do. This distinction has retained analytic purchase subsequently (e.g. StIrling, 2008). While the different justifications for engaging are not always as clear in practice as such a typology suggests, typologies can nonetheless highlight how different positions sit in tension to each other.

The proliferation of different ‘engaged’ health research modalities, and the contested conceptual landscape currently surrounding public engagement, raise the quite simple questions of what, exactly, is going on here, and how should we navigate and make sense of this melee of concepts and practices? In this paper, we, an interdisciplinary group of social science and humanities scholars working within health-related research, build on our different disciplinary perspectives and experiences to set out what we see as important, outstanding challenges around the practice and theory of engaging. The paper was generated through many discussions and collective writing, motivated by our shared concern around the manifestations of engaging in a context where public engagement has been persuasively mainstreamed. This has occurred despite the lack of consensus highlighted above, and the underplayed epistemic dimensions, power relations, and varied value frames that characterise engaging. Our aim here is to move beyond questions of terminology to consider the tensions and possibilities that the diverse landscape of engaging evokes; we will not create another typology or argue for the use of specific terms over others. To signal this attempt at synthesis that challenges and moves us forward, we will use the term ‘engagements,’ both for practical reasons, as an umbrella concept that can capture the plural, interlaced and overlapping practices that circulate in this space. ‘Engagements’ function as ‘problem concept’ that can be worked with yet has many uses and no settled meaning (see also Parry *et al.*, 2012). Notwithstanding the conceptual and practical ambiguity of the object, there is analytic value in considering the broad range of engagements together, allowing reflection on the intrinsic tensions, discomforts, and possibilities around doing and researching engagements.

In what follows, we consider the roots, present modalities and institutional frameworks that have been erected around engagements, including how these shape and delimit how engagements are framed, enacted, and justified. We also consider the implications of this for developing alternative practices and frameworks that could enable flexibility and contextually relevant diversity. We examine the related issue of knowledge production within and through engagements, addressing whether engagements can, or should, be framed as knowledge producing activities, what kinds of knowledge they might generate, and who is (and is not) positioned as a knowledge producer, within the confines of wider power relations and epistemic hierarchies that constrain the roles that can be attributed to different actors. We then unpack how engagements are or could be valued and evaluated, emphasising the plural ways in which ‘value’ can be conceptualised and generated by different actors involved in engagements, and how this plurality limits the ability to fix or measure value from engagements.

We conclude by calling for a philosophy of engagements that can capture the diversity of related practices, concepts and justifications around engagements, and account for the plurality of knowledges, knowledge producers and kinds of value that engagements engender, while remaining flexible and attentive to the structural conditions under which engagements occur (see also Mockford *et al.*, 2012; Rowland *et al.*, 2017). We argue that this philosophy should be a feminist one, informed by feminist epistemological and methodological approaches to equitable modes of research participation, knowledge production, and valuing. This will enable a synergy of empirical, epistemic and normative considerations in developing accounts of engagements in both theory and praxis. Modestly, we hope to carve out the starting points for this work.

## The roots, manifestations, and mainstreaming of ‘engaged research’

The current landscape of engagements within health-related research has been significantly crafted by changing social and institutional contexts, policies, and wider questions of research governance. Among the most influential developments have been the changing relationship between doctors and patients, partially driven by patient and health advocacy and activist movements since the 1960s, including the women’s liberation health movement, HIV/AIDS activism and disability and mental health movements. These have all demanded and provided access to and created their own health knowledge, giving rise to some of the first ‘expert patients,’ as patients and activists became disillusioned with professionals’ ability to treat them and the control exerted over the definition and management of their bodies, health and illness by healthcare professionals ( Berghs *et al.*, 2020; Epstein, 1996; Murphy, 2012; Rosenberg & Rosenberg, 2018). This sparked a shift in the relationship between patients and healthcare organisations as well as between research participants and research institutions, as patients and participants increasingly demanded recognition of their agency and rights, including to participate in healthcare and health research decision-making. Another influential shift has been that towards transparency in having publics involved in governance and ethical decision-making, including following medical malpractice cases and in the development of controversial technologies that raised questions about, and undermined, public trust in science. For example, IVF techniques in the 1980s incited public debate in the UK about the responsibilities that scientists and science itself (should) have towards society. This served to catalyse increased involvement of publics in science governance and public debate on who should have the authority to make decisions about ethical questions raised by science in a context of disagreement and value pluralism ( Moore, 2010).

Developments like these constitute a background against which engagements have become increasingly mainstreamed, and institutionalised. Today, patients and publics are participating in health research and healthcare in more diverse ways than before through mandates for engaged research, beyond being (merely) recipients of information and services from health professionals. For example, the UK National Institute for Health Research (NIHR) has made ‘patient and public involvement’ a requirement for research funding, with other funders following suit. Recently, the NIHR, among others, have also begun to use the term ‘co-production,’ entailing a conceptual shift towards more egalitarian and collaborative models as an avenue for improving health research engagements ( NIHR, 2015). The NIHR has been influential in defining, through guidance and policy, what constitutes ‘good’ engagements, especially through their INVOLVE programme, which initially supported engagements in the National Health Service (NHS). INVOLVE (2012) published resources setting terms of ‘good practice’ in engagements, including guidance for researchers on how to involve publics. Other organisations, such as the Jefferson centre in the US also define ‘best practice’, in this case in relation to the approach to citizens juries that they promote ( Jefferson Centre, 2020). More generally, engagements are increasingly tethered to institutional practices, including universities setting up their own structures and processes, guided and supported by the National Co-ordinating Centre for Public Engagement. The University of Edinburgh where we work, for example, is involved in several networks around engagements, including the Beltane Public Engagement Network ( Beltane, 2020), the Scottish Public Engagement Network ( ScotPEN, 2020), as well as its own structures of professional public engagement support.

The notions and frameworks of ‘good’ and ‘best’ practice promoted by different organisations are often credentialed modes of knowledge (coming from and legitimated by authoritative institutions) and advocated either implicitly or explicitly as the ‘right’ knowledge. Notably, they tend also to be delineated in ways that align with the institutions’ broader objectives and agendas. For example, drives to improve engagements, including ‘good’ or ‘best’ practice frameworks in the UK, have coincided with recommendations to measure success from engagements in the context of the Research Excellence Framework (REF). REF assesses research quality including an evaluation of its impact, which is defined in terms of the effects, changes, or benefits achieved ‘beyond academia’ ( REF2021, 2019). In this context, engagements are being framed not only in terms of more egalitarian or collaborative research, but also as a way to realise research impact, often as defined in the REF ( Paylor & McKevitt, 2019; Smith *et al.*, 2020). This may be a cause for caution both around the underlying motivations that drive institutional engagements and what the promotion of engagements ‘does’ in practice ( Paylor & McKevitt, 2019; Smith *et al.*, 2020).

Indeed, the aims, justifications and motivations for engagements are often unclear in this context: shifts towards ‘co-production’ advanced by some organisations may be formally justified by substantive aims of improving research through such engagements, or normative aims of enabling more egalitarian and collaborative research, yet the practical motivations that foreground engagements may not necessarily match the rationales that are formally used to justify them ( Esmail *et al.*, 2015). For example, Paylor & McKevitt (2019) have argued that a form of ‘authoritative instrumentalism’ underpins the NIHR’s and others’ shift to ‘co-production,’ where engagements are seen as something to be implemented to deliver particular kinds of instrumental outcomes. This is precisely because ‘co-production’ has gained institutional currency in the context of policy initiatives highlighting and prioritising the impact of research outside academia (see also Williams *et al.*, 2020).

Similar tensions, shaped by the institutionalisation of engagements, can also extend to local practices of engagements undertaken ‘on the ground’ within research projects. While the formal rationale for engagements within research projects may be normative or substantive motivations to ‘do the right thing’ or improve research outcomes, the practical motivations driving engagements may be more instrumental, shaped by institutions’ and research funding bodies’ requirements for incorporating engagements within research ( Paylor & McKevitt, 2019). The institutionalised framework through which engagements are mandated can appear monopolistic, unhelpfully closing down debate and space for appreciating the ambiguities around engagements, when practices are still being developed and should be reflected upon and questioned. As Paylor & McKevitt (2019) have suggested, engagements can become an impoverished sphere of activity where researchers are undertaking them because it is a funding requirement without the opportunity to reflect more fully on why. Moreover, mainstream institutionalised models of ‘good’ or ‘best’ engagement practice may at times have the effect of quietening down alternative ways of thinking about engagements or side-lining them in such a way that alternative spaces and activities come to be understood in opposition to mainstream models. This is especially so when models of and claims to ‘good’ or ‘best’ practice are transported into new contexts, in ways that can make it more difficult to diversify and develop alternative engagements that would better account for cultural and local differences.

For example, ‘patient and public involvement’ is now a requirement of joint funding through the UK Medical Research Council (MRC) and NIHR Global Health partnerships in Low- and Middle-income Countries (LMIC). The framework that has been applied, however, raises questions around whether it is appropriate to take models for engagements developed in High-income Countries (HICs) and directly apply them in LMICs, especially without building on the experiences from research undertaken in these countries as their usefulness may be limited due to local differences. These differences can include structural barriers to participation in public involvement initiatives such as physical access, poverty, and social or cultural exclusion of different marginalised groups in many settings (e.g. Bolsewicz Alderman *et al.*, 2013). Indeed, it may seem that engagement activities are being absorbed by large HIC institutions, leaving limited space for alterative conceptualisations and enactments of engagements, including the development of culturally and locally appropriate frames. The Global Challenges Research Fund (GCRF), however, now requires equitable partnership building with LMIC partners as a way to address challenges around HIC-LMIC power relations in research contexts ( UKRI, 2020), also increasing opportunities for local engagement frameworks. It remains to be seen how these local frames develop in the new partnerships that are being forged.

These issues are connected with other limitations for realising engagements, especially co-production, via institutionally funded academic research. While there are exceptions (c.f. Collins *et al.*, 2020), decision-making about research questions and design, for example, still tends to remain in researchers’ hands. Publics or patients are usually involved with more limited questions like design of patient information sheets, and typically only on a study-by-study basis, constraining temporally sustained engagements with research more broadly conceived ( Paylor & McKevitt, 2019). Also, despite the increasing availability of guidance documents on how to conduct engagements, the creation of engagement mandates has not led to the formalisation of training for researchers and healthcare professionals in the skills to carry out ‘good’ engagements. This has facilitated the commercialisation and professionalisation of engagements where ‘independent’ engagement practitioners and organisations are sometimes employed to undertake engagements on researchers’ behalf (see Bherer *et al.*, 2017; Pallett, 2019). Yet, professional roles to carry out engagements, within both universities and independent professional engagement agencies, may not have clear career development pathways, leading to a situation where skilled and experienced engagement professionals may need to leave these roles to progress, limiting the learning and experience that can be achieved. This is especially problematic because much of the relevant knowledge is gained through experience, making the informal as well as formal sharing of experiences important for learning from, and improving, engagements.

What is needed in this context, then, is reflexivity both about the contextually conditioned nature of mainstreamed, institutionally authorised models of engagements, and about the actual motivations and stated justifications around engagements that are being deployed within the confines of institutionalisation. The mainstreaming of engagements has opened positive, potentially empowering spaces for bringing new actors and voices into science, which can challenge conventional epistemic and power hierarchies in science governance. However, institutionalising engagements carries the danger of homogenising and ‘fixing’ engagements into particular, authorised models of ‘good’ or ‘best’ practice. This may problematically silence alternative ways of imagining and enacting engagements, and constrain the critical space left for dynamism. We call for critical, theoretical and practical reflection on the models, motivations, and justifications based on which engagements are currently undertaken. Such reflection must connect with the relationship between engagements and knowledge production to which we now turn.

## Engagements and knowledge production

The institutionally shaped nature of engagements raises questions about engagements’ epistemic dimensions: what do engagements, at different moments of the research process, do to processes of knowledge production? To what extent are engagements knowledge producing activities, and should they be? What sorts of knowledge might be produced and shared, by whom, for whom, and for what purposes? These questions are centrally connected with the value and positions attributed to different types of knowledge and knowledge producers within engagements and research processes.

Institutional framings and structures around scientific knowledge production tend to delimit what counts as knowledge producing activity, and the types of knowledge that can be generated. Firstly, research governance frameworks often explicitly differentiate between research and engagements, where the former is, for example, subject to ethical oversight while the latter may not be. While being exempt from oversight procedures can be liberating in permitting creativity and experimentation, it can limit what knowledge engagements are able (or allowed) to generate. There are difficulties converting insights from engagements into valid(ated) knowledge, for example, if the engagements have not undergone the institutional ethical review process required for research with human participants, which presents a barrier to publishing findings especially in peer-reviewed journals. Engagements undoubtedly generate knowledge, but when the epistemic orientations surrounding these practices fail to recognise the value of this knowledge and institutional structures are not conducive, it may not be captured in a usable way, limiting the possibilities for learning from engagements. This raises questions around whether an unequal relationship with respect to knowledge is always implicit in mainstream conceptualisations of engagements: is the differentiation between research and engagement being made via an equation of research with (valid) knowledge production, and relegation of engagements as not (valid) knowledge producing activities? How are these boundaries drawn, why, and by whom?

Secondly, institutional framings and structures around scientific knowledge production tend to delimit, either explicitly or implicitly, the kind of roles that differently positioned actors can occupy in relation to knowledge; i.e. who ‘counts’ as or is permitted to be a knowledge producer. As feminist theorists, among others, have long argued, mainstream scientific epistemologies have privileged forms of knowledge production that (claim to) transcend contextual differences and the particularities of subjective experiences, even though all (including scientific) knowledge arises from particular perspectives in ways that make it context-dependent and value-laden (e.g. Alcoff & Potter, 1993; Antony, 2018; Grasswick, 2011). The ‘expert’ perspective of trained career scientists has, however, been universal *ised,* presented as objective, and as paradigmatic of (valid) ‘knowledge,’ with the effect that the knowledges of non-scientists, and especially knowledge arising from subjective experience, have been positioned as not knowledge in the proper sense; ‘improper knowledge.’ Relatedly, various types of engagements often implicitly position certain actors as holders or producers of knowledge, while others are framed as receivers or as sources of data.

This raises the epistemic issue of positionality (i.e. how people are positioned and position themselves) in knowledge production processes. In recent decades, the epistemic privilege of the conventional ‘expert’ perspective has been challenged including through the emergence of what have been termed ‘professional layperson’ and ‘lay expert’ roles. Those occupying these roles are generally defined in opposition to (conventional) ‘experts’ but are also, by the very nature of their role, taken to possess a particular expertise: positional experience and ability to navigate the engagements environment and effectively provide ‘lay perspectives’ that are expected and looked-for within these contexts ( Kerr *et al.*, 2007). A further twist on these roles occurs when academics (e.g. bioethicists or social scientists) or professionals with expertise in healthcare and research are cast as ‘laypersons’ and therefore (expected to) represent ‘lay perspectives’ in relation to health-related knowledge in engagement contexts ( Kerr *et al.*, 2007). Similarly, the notion of expertise-by-experience is now commonplace across a range of health governance contexts, but can also generate conflicted, confusing, and sometimes impossible roles for participants ( Meriluoto, 2018). This also raises potentially difficult questions about the extent to which having a particular epistemic standpoint (e.g. patienthood and, as an effect of that position, expertise on illness through experience) means that a person knows ‘more’ or ‘better.’ Inhabiting a particular social location does not necessarily mean that the knowledge arising from that location is more valuable than other, differently located knowledges ( Wylie, 2003).

Further related questions are raised by approaches to research that promise more active roles for participants, including ‘co-production’ or participatory research. For example, some have considered whether research participants should be included as co-authors on academic outputs, to recognise the often direct and invaluable contributions they make to knowledge production, and the challenges around doing this in practice within academic publishing structures (c.f. Bain & Payne, 2015). An example of a related innovative approach is the Frontiers for Young Minds journal, reviewed by a board of children and teenagers with the aim of enabling young people and scientists to work together to create academic articles ( Frontiers, 2020). This is also connected with the issue of how participants’ ‘lived experience’ should be situated within academic environments ( Banfield *et al.*, 2018). Participants’ role in research, as more than mere sources of data, may demand that they should be recognised as knowledge producers, but incorporating these non-conventional actors into the existing academic hierarchies can provoke perhaps uncomfortable questions about the purpose and worth of academic training and legitimacy of academic credentials. We can also ask whether recognition through co-authorship is the format that would be most meaningful to participants, or whether this mode of recognition is, itself, built on the priorities of academics. Indeed, there may be a need to develop different modes of recognition beyond the academic epistemic and value frameworks.

We contend that feminist methodological tools and theoretical approaches (c.f. Fonow & Cook, 1991; Ramazanogly & Holland, 2002) can help with the above kinds of challenges. While related bodies of feminist theory are diverse, reflecting the plurality of feminisms more generally, there are some widely shared epistemic tendencies within this diversity and plurality that offer avenues for thinking differently about the epistemic tensions around different kinds of engagements. Feminist epistemologies and methodologies have long sought to destabilise traditional power differentials between researchers and research participants by exposing and reclaiming experiences as epistemically salient, often positioning subjective experiences (especially of marginalised groups) as key to knowledge production. They have emphasised the situatedness and contextuality of all knowledge claims, beginning from the assumption of knowledge plurality and equity, while conceptualising sound knowledge production in general as requiring dialogical engagement with this plurality and partiality. This demands reflexivity from those positioned as ‘experts’ on what shapes their own epistemic starting points and interpretations ( Code, 1988; Haraway, 1988; Harding, 1986; Harding, 1991).

Applying this to engagements entails recognition and endorsement of the fact that there are potentially multiple sets of knowledge at play within any given engagement – not (just) singular ‘lay’ and ‘scientist’ but plural knowledges and positions from which knowledge is generated, including those of facilitators, practitioners, funders or commissioners, etc. – and that knowledge progresses dialogically. Conceptualising engagements centrally as practices of knowledge production focuses attention both on the epistemic processes and end products, implying that our valuations of engagement should orient around how different knowledge is generated through engaged research and by whom. These issues are also connected with the question of how and why engagements are valued and evaluated.

## Valuing and evaluating engagements

As practices and processes of engagements have been mainstreamed, calls for evaluation of engagements have also grown (c.f. Oliver *et al.*, 2019) but the processes of evaluating are often not well documented in existing literature ( Esmail *et al.*, 2015). Formal mechanisms for evaluating engagements are, however, increasingly required by funding bodies and institutions, and expected by other stakeholders. These may include qualitative, quantitative, or mixed approaches to develop understanding of individuals’ and groups’ perceptions of engagements, increase awareness, or improve participation rates. The UK Research and Innovation (UKRI) evaluation guide, for example, lists evaluation techniques based on social and market research methods like surveys, interviews, focus groups and discussions with target groups or wider publics ( UKRI, 2011). Yet, the formalisation of evaluating engagements raises further questions around how and why evaluations are undertaken, to whose benefit, whose views are represented and whose may be excluded.

Formal evaluation guidelines often define evaluation, implicitly or explicitly, in terms of determining and improving the quality and impact of engagements, where ‘impact’ is often framed in relation to effects, changes, or benefits ‘beyond academia,’ as delineated in the REF. The UK MRC (2020), for example, describes evaluation as something that is done by measuring the learning or changes in thinking that those who take part in engagement activities gain from these activities, and by measuring their inspiration to know more or get involved in further activities. The information that is collected through evaluation is usually seen as a means to increase the value of engagement activities, while ‘value’ is associated with ‘impact.’ Further, models of evaluating engagements tend to draw from evidence-based intervention models, where engagements are seen as akin to an intervention to be evaluated. Conceptualising engagements as interventions or tasks with pre-defined objectives to fulfil, however, does not fully capture the inherently human aspect of engagements, including relationships, exchange of knowledge and ideas that often results in profound and insightful experiences for those involved ( Komporozos-Athanasiou *et al.*, 2016). Institutional framings of evaluation can have the effect of not only delimiting what (proper) ‘evaluation’ should look like, but also pre-defining the purpose of evaluation as impact and quality assessment, in ways that can silence the potential of evaluation exercises to produce valuable insight and knowledge, in their own right.

Indeed, what the purpose or ‘value’ of engagements’ evaluation is or should be, what kinds of activities and methods (should) count, legitimately, as ‘evaluation,’ and for whom has been contested, as has the distinction between ‘evaluation’ and ‘research.’ Purtell *et al.* (2012), for example, have critiqued the drive to emphasise impact and measurement of engagements without due reflection on the purpose or rationale of engagements in the first place. Boivin *et al.* (2018), moreover, found that publics are often not involved in the development and design of the evaluation tools in the first place.

Feminist approaches to evaluation, on the other hand, have applied alternative modes and frameworks of (e)valu(at)ing. These include empowering, multi-vocal and appreciative inquiry methods which aim to elucidate social inequalities and increase the capacity of individuals and groups to effectively represent their views on both engagements and research activities through evaluation (c.f. Patton, 2002). Some have stressed how individuals from different social backgrounds approach information and evaluation processes in different ways, showing not just that impact has (or has not) occurred, but also why and how people are affected, how they interpret, perceive, and, indeed, value information in the context of their own lives ( Sielbeck-Bowen *et al.*, 2002). Action-based paradigms have sought to link the results of evaluations with wider questions of social justice, by mobilising evaluation findings to address structural and procedural processes through which some voices and forms of knowledge are prioritised at the expense of others ( Mertens, 1999). This requires rethinking both evaluation and engagements as forms of action that simultaneously assess, inquire, and aim to act upon inclusion and exclusion processes within research, engagement and evaluation processes.

These approaches highlight that evaluation may serve multiple purposes, including social, cultural, political and financial; and, that the ‘value’ of engagements is less an intrinsic singular property and more something that is multi-directional and actively created through engagements and evaluation processes. Moreover, different ideas about the purpose and value of engagements and evaluation may also conflict, as different groups and individuals might prioritise differently (also leaving open the possibility that some engagements may not have value to any participants). For institutions, engagements may carry value in their potential to improve research quality and produce more accessible outputs and opportunities for knowledge sharing, but they also carry symbolic and financial value in showcasing research ‘impact’ beyond academia, and increasing success in research funding. Value may also be gained, however, by those taking part in engagements, for example through offering a sense of purpose to someone who faces life disruptions as a consequence of disease or illness, or the value in creating and fostering relationships between others who are engaging and also with researchers ( Komporozos-Athanasiou *et al.*, 2016). These kinds of value may be hard to measure, or immaterial in substance, despite being important to those engaging.

These different forms of value are shaped and constrained by the wider social and structural contexts in which they are generated, and in which research and engagements occur. Systemic structural inequalities, including along the lines of gender, race, class, (dis)ability, sexuality, immigration status and other socially significant differences simultaneously embed the institutional contexts in which research and engagements are undertaken, and delimit who participates (and does not) in engagements, whose voices are heard and views represented in evaluations ( Sielbeck-Bowen *et al.*, 2002). These structural forces – both of the funding and political landscapes, and the wider societal structures that embed them – are not always stabile, creating challenges in obtaining consensus, and leading to changing emphasis and value in engagements around who should be engaged with, why, and what it means to engage. Interesting critical questions are also raised when the symbolic value gained by institutions is considered. Boylan *et al.* (2019) have provided insight into the identities of scientists for whom responsibility for engagements is assumed: early-career researchers, postgraduate students and, often, women. Such disparities in gender and career hierarchy raise additional concerns around how engagements may or may not be valued in ways that reflect wider epistemic and gender hierarchies. These include the mainstream scientific epistemic paradigm that has simultaneously devalued, gendered (as ‘women’s work’), and positioned as epistemically inferior, social scientific and humanities approaches to producing knowledge, including the methods primarily used in evaluation of engagements.

Engagements should be conceptualised in ways that are grounded in acknowledgement and analysis of the array of different kinds of value that they can embody and generate. Simultaneously they should recognise that one cannot fully measure whether, how and for whom engagements have had value without reflecting on the processes and justifications of engaging, and the wider social structural conditions under which they occur. Engagements serve multiple purposes and result in multiple kinds of value for all actors involved in the activities. Thus, we advocate for an approach that builds on this insight to centre the question of value independent of its capacity to be fixed and measured, to understand and explicate how the value of these processes is plural. Building on feminist epistemological frames and approaches to (e)valu(at)ing enables active concern both for the plural ways in which ‘value’ manifests, and for how, why, and what kinds of information are produced through evaluation, as well as how this information is epistemically positioned in relation to research and ‘knowledge.’ Variation in how engagements are evaluated is, notably, not intrinsically negative, but it is necessary to identify and reflect on tensions and discomforts that arise when different forms of value are prioritised, and to ask what value is obtained, by whom, and what social and structural conditions shape this.

## Conclusion: towards a feminist philosophy of engagements

This paper has aimed to set out what we see as important outstanding questions and issues around engagements, especially in the UK context but also beyond. We have used the notion of ‘engagements’ as a problem concept that can nonetheless capture the plural and overlapping ways in which engagements are enacted. We move beyond attempts to delineate definitions or the scope of what does or should count as engagements, in order to focus on the tensions and possibilities that the multiplicity of engagements evoke.

In considering the roots, current manifestations and mainstreaming of engagements especially within institutional contexts, we argue that there is a need to remain reflexive about how these contexts condition the possibilities for engagements, and we call for critical inquiry into the models, motivations and justifications through which engagements are currently enacted. We are concerned with engagements’ epistemic dimensions, especially questions around how engagements are situated in relation to knowledge production. Thus, we call for ongoing inquiry into the kind(s) of knowledge engagements can or should produce, how this knowledge is or should be captured, who is and is not positioned as a knowledge producer, and what kinds of power relations condition the roles attributed to different subjects. We are also concerned about how engagements are and could be evaluated by different actors, to whose benefit, and how ‘value’ is being conceptualised in these processes, and call for recognition of the diverse kinds of value that engagements can bring to different actors involved, including how people can be and are affected by engagements. This inevitably entails understanding ‘value’ as plural, limiting its capacity to be fixed or measured.

In conclusion, we argue that there is a need for a ‘philosophy of engagements’: one where ‘philosophy’ entails inquiry into what kind of activity something is and how we should do it, and where the term ‘engagements’ remains problematised but necessarily plural, to leave space open for the creative potential of engagements for exploring new approaches that can enable us to re-think what engagements are, could be, and how to do them. We argue that this philosophy should be a feminist one, informed by feminist approaches to social power relations, knowledge production, value generation, and their relationship. This is because translating existing feminist theoretical and methodological tools into the context of engagements, can help us to address the central outstanding questions and tensions that we have found through our work and experience around engagements.

Firstly, a feminist philosophy of engagements should centre the feminist notion of reflexivity as a key tool in designing and enacting engagements, because this can enable one to critically interrogate the epistemic starting points and presumptions that shape how engagements are conceptualised and undertaken, and how these starting points direct the kinds of knowledges that can (and cannot) be produced through them, especially within institutional confines. Actively practicing reflexivity can also enable us to shed critical light on the contextually conditioned nature of institutionally authorised models of engagements, and identify and address possible tensions between different motivations and stated justifications around engagements. We should embed reflexivity into how we conceptualise engagements, and how and why we enact them, because this can enable us to not only acknowledge how institutional pressures and predefined ‘best practice’ models may influence our own practices and motivations for undertaking engagements, but it can also enable us to challenge these pressures and models to develop alternative ways of imagining and enacting engagements. Doing so is especially pertinent when understood in the wider context where systemic structural inequalities and hierarches delimit the practical realities of who is included in (and excluded from) engagements. Thus, we recommend reflexivity as both a conceptual and practical tool to develop critical analysis of existing engagement models and alternative frameworks of engagements.

Secondly, and relatedly, a feminist philosophy of engagements should centre feminist notions of positionality as this can facilitate critical interrogation of knowledge production both in research and engagements, including in relation to (e)valu(at)ing engagements. In conceptualising, designing and enacting engagements, we should start with the feminist insight that all knowledge, including scientific knowledge, arises from a perspective that is shaped by the knowledge producers’ social location. This insight leads to an understanding of knowledge as situated, plural and partial, which is turn will enable us to ask critical questions about who are (and are not) recognised as knowledge producers, and which knowledges are (and are not) valid(ated) in research and engagements. These questions should include interrogation of the very distinction between research and engagements as separate(d) activities, and the extent to which we should see them as such. We recommend applying the tool of positionality to understand engagements because it can enable us to recognise, unpack, and name the active roles that many different actors play in the generation of knowledges from research and engagements, and the plural kinds of value attributed to them. Reversely, it can also enable us to identify which positionalities and knowledges are not being represented. By emphasising positionality in all knowledge production, we can begin to destabilise conventional epistemic and power differentials between researchers and research participants and expose participants’ knowledge as epistemically salient and valuable.

A feminist philosophy of engagements advanced along these lines would necessarily focus centrally on the processes as well as end products of engagements, unpacking how engagements serve multiple purposes, generate plural knowledges, and carry manifold kinds of value that require recognition and critical interrogation. Notably, the above outlined issues are characterised by the intertwining of empirical, epistemic and normative questions around engagements. Thus, a feminist philosophy of engagements will inevitably require dialogical engagement with this multiplicity and plurality via interdisciplinary modes of thinking, to facilitate the concurrent integration of the empirical, epistemic, and normative spheres in developing accounts of engagements both in theory and praxis. This paper has aimed to carve out the starting points for this work, showing how feminist perspectives offer avenues for thinking differently about engagements, within and beyond institutionalised models.

## Data availability

No data are associated with this article.
