# Towards a feminist philosophy of engagements in health-related research

**DOI:** 10.12688/wellcomeopenres.16535.2

**Published:** 2022-02-10

**Authors:** Sonja Erikainen, Ellen Stewart, Angela Marques Filipe, Sarah Chan, Sarah Cunningham-Burley, Sophie Ilson, Gabrielle King, Carol Porteous, Stephanie Sinclair, Jamie Webb

**Affiliations:** 1 Centre for Biomedicine, Self and Society, Usher Institute, University of Edinburgh, Edinburgh, EH8 9AG, UK

**Keywords:** engagement, involvement, health research, feminist, epistemology, evaluation

## Abstract

Engagement with publics, patients, and stakeholders is an important part of the health research environment today,and different modalities of ‘engaged’ health research have proliferated in recent years. Yet, th ere is no consensus on what, exactly, ‘engaging’ means, what it should look like, and what the aims, justifications, or motivations for it should be. In this paper, we set out what we see as important, outstanding challenges around the practice and theory of engaging and consider the tensions and possibilities that the diverse landscape of engaging evokes. We examine the roots, present modalities and institutional frameworks that have been erected around engaging, including how they shape and delimit how engagements are framed, enacted, and justified. We inspect the related issue of knowledge production within and through engagements, addressing whether engagements can, or should, be framed as knowledge producing activities. We then unpack the question of how engagements are or could be valued and evaluated, emphasising the plural ways in which ‘value’ can be conceptualised and generated. We conclude by calling for a philosophy of engagements that can capture the diversity of related practices, concepts and justifications around engagements, and account for the plurality of knowledges and value that engagements engender, while remaining flexible and attentive to the structural conditions under which engagements occur. Such philosophy should be a feminist one, informed by feminist epistemological and methodological approaches to equitable modes of research participation, knowledge production, and valuing. Especially, translating feminist tools of reflexivity and positionalityinto the sphere of engagements can enable a synergy of empirical, epistemic and normative considerations in developing accounts of engaging in both theory and praxis. Modestly, here, we hope to carve out the starting points for this work.

## Disclaimer

The views expressed in the article are those of the author(s). Publication in Wellcome Open Research does not imply endorsement by Wellcome.

From patient involvement panels to twitter chats to citizens’ juries, public engagement is part of the health research environment today, in the UK and beyond. The terms ‘engagement’ and ‘involvement’ are part of a spacious and expansive conceptual landscape where multiple terms are used simultaneously and in overlapping ways across academic, policy and public discourses. Sometimes, different terms are used interchangeably to capture practices that follow different, even contrasting logics ( Duschinsky & Paddison, 2018), including ‘public,’ ‘patient,’ ‘community,’ or ‘stakeholder’ ‘participation,’ knowledge ‘communication,’ ‘exchange,’ and ‘co-production.’ While the different terms collate diverse practices, mechanisms and communities to change the relationship between science and wider society, it is no longer considered acceptable for health research to be ‘un’-engaged.

There is no consensus on what, exactly, ‘engaging’ means or should look like, so we need critical interrogation of the concepts and practices around engaging (see e.g. Crowe *et al*., 2020; Stilgoe *et al*., 2014). Different terms frame the people who are to be engaged with, and their roles, in different ways. There are not merely labels, but act to shape practices of engaging, including, for example, whether people are ‘involved’ in, ‘engaged’ with, or ‘co-producers’ of research. While many terms centre the ‘public’ as those who are engaged with, others focus on specific groups such as ‘patients.’ There has also been a shift away from the notion of a singular, undifferentiated public with a unified perspective (e.g., beyond the notion of a singular ‘patient perspective,’ see Rowland *et al*., 2017), towards a recognition of publics as plural, acknowledging diversity (see Felt & Fochler, 2010). Concurrently, despite the multiplicity of terms used to describe engaging and engagement, those who are doing the engaging are rarely explicitly specified. Rather, their identities remain implicit yet presumed, i.e. scientists, health professionals, researchers, and, increasingly, dedicated public engagement practitioners. Yet a distinction is generally drawn between the ‘engagers’ and the ‘engagees’ as the two sides in the science/society relationship. The central role of public engagement practitioners who often plan and undertake the engaging, is notably silent across these conceptualisations.

There are additionally competing accounts of the aims, or motivations of engaging. One influential account from Fiorino (1990) distinguished between substantive rationales, where engagements are justified because they lead to better ends through dialogue; instrumental rationales, where engagements are undertaken to achieve some other pre-defined end, such as ‘better trust in science’; and normative rationales, where engagements are justified because they are the ‘right thing’ to do. This distinction has retained analytic purchase subsequently (e.g. Stirling, 2008). While the different justifications for engaging are not always as clear in practice, typologies can nonetheless highlight how different positions sit in tension to each other.

Our aim in this paper is not to resolve the above challenges, create another typology nor to argue for the use of specific terms over others. We do not aim to delimit what does or does not (or should or should not) count, legitimately, as ‘engaging.’ Rather, we hope to consider the tensions and possibilities of the diverse landscape of engaging. We use the term ‘engagements,’ both to signal this goal, and for practical reasons, as an umbrella concept that can capture the plural, interlaced and overlapping practices that circulate in this space. ‘Engagements’ in this paper functions as ‘problem concept’ that can be worked with yet has many uses and no settled meaning (see also Parry *et al*., 2012). While this may result in a loss of specificity, we contend that there is analytic value in considering the broad range of engagements together, allowing reflection on the intrinsic tensions, discomforts, and possibilities around doing and researching engagements.

The proliferation of different ‘engaged’ health research modalities, and the contested conceptual landscape currently surrounding public engagement, raise the quite simple questions of what, exactly, is going on here, and how should we navigate and make sense of this melee of concepts and practices? In this paper, we, an interdisciplinary group of social science and humanities health scholars, build on our different disciplinary perspectives and experiences to chart what we see as important, outstanding challenges around the practice and theory of engaging. We consider the roots, present modalities and institutional frameworks that have been erected around engagements, including how the mainstreaming of engagements can shape and delimit how engagements are framed, enacted, and justified. We examine the related issue of knowledge production within and through engagements, addressing whether engagements can, or should, be framed as knowledge producing activities, what kinds of knowledge they might generate, and who is (and is not) positioned as a knowledge producer, within the confines of wider power relations and epistemic hierarchies that constrain the roles that can be attributed to different actors. We then unpack how engagements are or could be valued and evaluated, emphasising the plural ways in which ‘value’ can be conceptualised and generated by different actors involved in engagements, and how this plurality limits the ability to fix or measure value from engagements.

 Our key aim is to make the case for a philosophy of engagements, where philosophy entails inquiry into what kind of activity something is and how we should do it. We call for a philosophy that can capture the diversity of related practices, concepts and justifications around engagements, and account for the plurality of knowledges, knowledge producers and kinds of value that engagements engender, while remaining flexible and attentive to the structural conditions under which engagements occur (see also Mockford *et al*., 2012; Rowland *et al*., 2017). We argue that the translation of existing feminist epistemological and methodological tools to equitable modes of research participation, knowledge production, and valuing into the sphere of engagements can enable the construction of precisely such a philosophy. Because of this, we start this paper with a consideration of the feminist tools of reflexivity and positionality, to embed our arguments within feminist epistemology and show how and why these tools provide a different les to engagements. We conclude that our proposed feminist philosophy of engagements can enable a synergy of empirical, epistemic and normative considerations in developing accounts of engagements in both theory and praxis. Modestly, here, we hope to carve out the starting points for this work. 

## Feminist reflexivity and positionality

While feminist theory is not a singular, but a plural entity composed of diverse (sometimes contradictory) theoretical lenses, perspectives, and commitments, it is nonetheless possible to draw out epistemic tendencies and theoretical tools that are widely (if not universally) shared across feminist scholarship. In doing so, we build on Ackerly & True (2008) to argue that a feminist-informed approach synergising theoretical insights across of feminisms can enable one to foreground considerations that can be applied by everyone, regardless of one’s relationship with feminist theory more broadly. For our purposes, these considerations are twofold and interrelated: feminist notions of reflexivity and positionality, both of which are derived from wider feminist epistemology.

Feminist epistemologies and methodologies have long sought to destabilise traditional power differentials within the processes of knowledge production (e.g. Alcoff & Potter, 1993; Code, 1988; Haraway, 1988; Harding, 1986; Harding, 1991; Hill Collins, 2000; Sandoval, 2000; Oakley, 2000). This includes the questions of how knowledge is produced, what counts as knowledge proper, and who is (allowed to be) a knowledge producer. Feminist scholars have challenged the proclaimed neutrality, objectivity, and epistemic superiority of scientific knowledge by showing how all (including scientific) knowledge arises from a particular and partial perspective. In science, this perspective has conventionally been that of a white, highly educated, middle-class European or North American male who occupies a professional role in science and builds knowledge based on positivist epistemology. Feminist epistemologies have shone light on how dominant epistemic paradigms privilege some knowers and ways of knowing while marginalising and silencing others, and they have highlighted and sought to subvert the power relations embedded in this process. This has included exposing and re-claimed subjective experiences as epistemically salient, often by positioning experiences (especially of marginalised groups) as key to knowledge production rather than as a hindrance to it.

Feminist notions of positionality arise out of these epistemological insights, emphasising the situatedness and contextuality as well as incompleteness of all knowledge claims. While there are multiple and differing feminist conceptualisations of positionality (see Nencel, 2014; Simandan, 2019), many build on Haraway’s (1988) notion of situatedness or ‘positioning’ as a key practice grounding knowledge ( Simandan, 2019). Positioning knowledge enables one to see the kinds of power (including social, institutional, epistemic, etc.) that enabled the making of the knowledge claims. Positioning entails making visible and interrogating the significance of one’s location within power relations – ‘positioned’ knowledge cannot claim universality but rather sees the world from a specific location, in ways that are explicitly particular and embodied ( Rose, 1997). Foregrounding positionality, then, entails starting from the assumption of knowledge plurality and partiality, while conceptualising sound knowledge production as requiring both ‘positioning’ and dialogical engagement with plurality and partiality.

 Feminist notions of reflexivity are intimately connected with positionality and have significantly influenced conceptualisations of reflexivity as a methodological tool more generally (see Lumsden, 2019). As with positionality, conceptualisations of reflexivity both within and beyond feminist theory are plural and contested ( Lumsden, 2019; Pillow, 2003; Slaney *et al*., 2019), but reflexivity as a means of accountability has been a key distinguishing feature of feminist epistemology ( Linabary *et al*., 2020). Feminist notions of reflexivity have especially emphasised the examination and interruption of power relations embedded in the processes and products of knowledge. They have centred around interrogating the positionality of the knowledge producer, including how the knowledge producer’s location within wider relations of power delimits the knowledge that is produced ( Linabary *et al*., 2020; Lumsden, 2019; Pillow, 2003). This includes interrogating and interrupting conventional epistemic hierarchies between researchers and participants, and ‘the knower’ and ‘the known.’ Feminist reflexivity has been framed as a way of doing research or producing knowledge differently: while reflexivity will not obliterate unequal power relations, it can highlight the epistemic starting points that constrain how we interpret the world, and it can foreground the messy and sometimes uncomfortable ways in which the knowledges we produce fail to be innocent of these power relations ( Pillow, 2003). 

 While the above is a selective outline of feminist theoretical tools, we contend that it offers a useful starting point to interrogate engagements. Feminist notions of reflexivity and positionality have mostly been conceived for and applied in the context of research. Transferring them into the context of engagements can enable a feminist philosophy of engagements.

Ours is not the first philosophy of engagements – indeed, several philosophies have been proposed and applied, perhaps most prominently including those derived from political philosophy and theories of democratic participation, such as deliberative democracy that form the basis of public engagement approaches like citizen juries and assemblies (see Delli Carpini *et al*., 2004; Webler & Renn, 2013). Others have mapped the multiple philosophical perspectives that have shaped approaches to engagements in science and technology studies (see Delgado *et al*., 2010; Durant, 2011). However, a philosophy grounded in feminist epistemology offers a productively different basis upon which to build engagements.

Our arguments and analysis were generated through many discussions within our Research Centre, motivated by our shared concern around engaging in a context where public engagement has been persuasively mainstreamed, despite the lack of consensus on definitional issues. Like all knowledge claims, however, our arguments and analysis arise from a particular perspective; namely, that of an international group of scholars and public engagement professionals, all but two of whom are women and all of whom have white privilege and institutionally legitimated positions in an interdisciplinary health research centre in a ‘Russell group’ university in an English-speaking high-income country. The knowledge that we strive to produce is also partial, shaped and constrained by who and where are located – epistemically, socially, and geographically. This paper, correspondingly, is not intended to generate final statements or universalisable basis for how to do or think about engagements, but rather, a situated and incomplete perspective that, we hope, can offer a productive provocation. 

## The roots, manifestations, and mainstreaming of ‘engaged research’

The current landscape of engagements with health-related research has been significantly crafted by changing social and institutional contexts, policies, and wider questions of research governance. While our intention here is not to provide a comprehensive genealogy of engagements, we wish to outline some key areas and shifts that we consider especially pertinent for making sense of engagements now.

Among the most influential developments is the changing relationship between researchers/doctors and patients, partially driven by patient and health advocacy and activist movements since the 1960s. For example, especially in HICs, the women’s health movement has since the 1960s demanded and provided women with access to knowledge about their own bodies and health when this knowledge had previously been the near exclusive purview of (primarily male) healthcare professionals ( Murphy, 2012). The HIV/AIDS activist movement in the 1980s gave rise to some of the first “expert patients,” as patient activists became disillusioned with medical professionals’ ability to treat them, and begun teaching themselves about medical science, conducting their own community-based research, and creating their own knowledge about the disease based on experience as well as science ( Epstein, 1996). Similarly, disability and mental health activists have challenged medical models of disability and mental health, and the control that medical professionals have exerted over the definition and management of their lives, arguing for their right to make decisions about their own bodies ( Berghs *at al*., 2020; Rosenberg & Rosenberg, 2018). These movements, together with other forms of patient activism, sparked a shift in the relationship between patients and healthcare organisations and research participants and research institutions, as patients and participants increasingly demanded recognition of their agency and rights, including to participate in healthcare and research decision-making.

In the context of regulation, a valuable example of how these shifting relationships shaped the research landscape is the case of ‘expanded access’ and the regulatory approval process in the US. Expanded access is the use of an as yet unlicensed medical product outside of a clinical trial for therapeutic benefit for serious or life-threatening conditions for which approved treatment options have been exhausted ( FDA, 2021). A milestone in the history of expanded access was the case of Josh Hardy in 2014, a boy with an aggressive form of kidney cancer, whose family requested access to brincidofovir, an investigational product produced by the biotechnology company Chimerix. While Chimerix initially refused this request, a public campaign led by Hardy’s family caused Chimerix, in collaboration with the FDA, to organise an additional open-label trial with Hardy as the first enrollee ( Moch, 2017). This case sparked both regulatory and practice shifts around expanded access in the US (see Caplan *et al*., 2018; FDA, 2020; Woollett & Jackson, 2017) and more proactive ethical reflection within the pharmaceutical industry regarding equitable expanded access – a development that engaged patient advocates have encouraged. Part of this has been the adage, adopted from the disability advocacy community, of ‘nothing about us without us’. Yet, closer relationship between industry and patient groups also carries risks. Patient advocacy groups can become co-opted as advocates for the interests of pharmaceutical corporations, pushing for their particular products to gain regulatory approval, even when clinical benefit has not been well established. For example, the Biogen Alzheimer’s drug Aduhelm was recently approved by the FDA through its Accelerated Approval regulatory pathways and with support from the Alzheimer’s Association patient advocacy group, which is partially funded by Biogen ( Ault, 2021). This was despite little evidence of the drug’s clinical benefit, following several failed trials, and despite critics arguing that its approval may harm patients, is expensive, and may direct resources away from more effective care (e.g. Ramachandran & Ross, 2021).

 These examples highlight the power of disruptive patient advocacy in regulation as well as knowledge production, but also how patient advocacy can be co-opted by traditional power structures, including commercial or market interests, in ways that may result in patients struggling to access expensive treatments with little evidence of efficacy. In the context of both commercially and publicly funded research, however, engaging patients and publics has been one way of addressing challenges of accountability and transparency. It may relate to particular forms of activism and ways of responding to broader social and political aims such as those of inclusion, social change, and health justice as is the case of rare diseases and developmental disabilities ( Filipe *et al*., 2021; Rabeharisoa *et al*., 2014).

 In universities, developments like the above constitute a background against which engagements have become increasingly mainstreamed and, consequently, institutionalised. Today, patients and publics are participating in health research and healthcare in more diverse ways than before through mandates for engaged research. For example, the UK National Institute for Health Research (NIHR) has made ‘patient and public involvement’ a requirement for research funding, with other funders following suit. Recently, the NIHR, among others, have also begun to use the term ‘co-production,’ entailing a rhetorical shift towards more egalitarian and collaborative models as an avenue for improving health research engagements ( NIHR, 2015). The NIHR has been influential in defining, through guidance and policy, what constitutes ‘good’ engagements, especially through their INVOLVE programme, which initially supported engagements in the National Health Service (NHS). INVOLVE (2012) published resources setting terms of ‘good practice’ in engagements, including guidance for researchers on how to involve publics. Other organisations, such as the Jefferson centre in the US also define ‘best practice’, in this case in relation to their approach to citizens juries ( Jefferson Centre, 2020). More generally, engagements are increasingly tethered to institutional practices, including universities setting up their own structures and processes, guided and supported by the National Co-ordinating Centre for Public Engagement. The University of Edinburgh (where we work), for example, is involved in several initiatives including the Beltane Public Engagement Network ( Beltane, 2020), the Scottish Public Engagement Network ( ScotPEN, 2020), as well as its own structures of professional public engagement support. 

The frameworks of ‘good’ and ‘best’ practice promoted by different organisations are often credentialed modes of knowledge (coming from and legitimated by authoritative institutions). Notably, they tend also to be delineated in ways that align with the institutions’ broader objectives and agendas. For example, drives to improve engagements, including ‘good’ or ‘best’ practice frameworks in the UK, have coincided with recommendations to measure success from engagements in the context of the Research Excellence Framework (REF). REF assesses research quality including an evaluation of its impact, which is defined in terms of the effects, changes, or benefits achieved ‘beyond academia.’ In this context, engagements are being framed not only in terms of more egalitarian or collaborative research, but also as an instrumental route to achieve research impact ( Paylor & McKevitt, 2019; Smith *et al*., 2020). This raises questions around the underlying motivations that drive institutional engagements and what the promotion of engagements ‘does’ in practice ( Paylor & McKevitt, 2019; Smith *et al*., 2020).

Indeed, the aims, justifications and motivations for engagements are often unclear in this context. Shifts towards ‘co-production’ advanced by some organisations may be formally justified by substantive aims of improving research through such engagements, or normative aims of enabling more egalitarian and collaborative research (see Filipe *et al*., 2017). Yet the practical motivations that foreground engagements may not necessarily match these rationales ( Esmail *et al*., 2015). Paylor & McKevitt (2019) have argued that a form of ‘authoritative instrumentalism’ underpins the NIHR’s and others’ shift to ‘co-production,’ where engagements are seen as something to be implemented to deliver particular kinds of instrumental outcomes. This is precisely because ‘co-production’ has gained institutional currency in policy initiatives highlighting and prioritising the impact of research outside academia (see also Williams *et al*., 2020), despite there being no consensus on what co-production means or entails ( Filipe *et al*., 2017).

Similar tensions, shaped by the institutionalisation of engagements, can also extend to the more micro sphere of engagements undertaken ‘on the ground’ within research projects. While the formal rationale for engagements within research projects may be normative or substantive motivations to ‘do the right thing’ or improve research outcomes, the practical motivations driving engagements may be more instrumental, shaped by institutions’ and research funding bodies’ requirements for incorporating engagements within research ( Paylor & McKevitt, 2019). As Paylor & McKevitt (2019) have suggested, engagements can become an impoverished sphere of activity where researchers are undertaking them because it is a funding requirement without the opportunity to reflect more fully on why. Moreover, mainstream institutionalised models of ‘good’ or ‘best’ engagement practice, while seeking to improve the quality and quantity of involvement happening, may in fact quieten down alternative ways of thinking, so that alternative spaces and activities come to be understood in opposition to mainstream models.

This is especially so when models of and claims to ‘good’ or ‘best’ practice are transported into new contexts, in ways that can make it more difficult to diversify and develop alternative engagements that would better account for cultural and local differences. For example, ‘patient and public involvement’ is now a requirement of joint funding through the UK Medical Research Council (MRC) and NIHR Global Health partnerships in low- and middle-income Countries (LMIC). The framework that has been applied, however, raises questions around whether it is appropriate to take models for engagements developed in HICs and directly apply them in LMICs without building on the experiences from research undertaken in these countries (see e.g. Bolsewicz Alderman *et al*., 2013). Indeed, engagement activities are being absorbed by large HIC institutions, leaving limited space for alternative conceptualisations and enactments of engagements, including frames based on local perspectives and needs. The Global Challenges Research Fund (GCRF), however, now requires equitable partnership building with LMIC partners as a way to address challenges around HIC-LMIC power relations in research contexts ( UKRI, 2020), creating opportunities for more appropriate, bottom up, local engagement frameworks.

Some health-related research in LMICs has grappled with the contested relationship between the priorities of researchers and the priorities of study participants. This has led to work that attempts to be genuinely participatory, co-led by local communities and driven by their needs, often carried out under the umbrella of ‘participatory action research (PAR)’ and, in some contexts, that of ‘community engagement’ which has otherwise been advocated in global health governance and research agendas (e.g., WHO, 2017). For example, local communities in rural South Africa have been engaged in identifying problems and developing action plans to secure clean drinking water ( Participedia, 2020a), and address under-five mortality ( Participedia, 2020b). In Nepal, participatory interventions led by women’s groups addressed poor birth outcomes for women throughout rural areas of the country – work which researchers attributed to a 30% decrease in neonatal deaths in the communities running these interventions ( Participedia, 2021). Comparatively, however, research-action methodologies and epistemologies from the South have remained far less visible to the engagement scholarship produced in European and North American contexts, even in scholarship seeking to “situate” research engagements and interventions ( Filipe, 2017). In South America and especially Brazil, for instance, there is long history of alternative approaches to engagement whose roots can be traced back to the critical pedagogies of emancipation and democratic participation developed by Freire (1996) and Boal (2000). Where more participatory and less institutionally directed research approaches have been applied within LMICs, there are also ongoing challenges for these approaches to have ‘impact’ in informing policy or secure adequate resources for long term, rather than one-off, projects. Attention to these alternative approaches can, however, highlight that the models of research and engagement that are prioritised and applied in institutional contexts (especially) in HICs are an active institutional choice and driven by power dynamics, including around what is considered an appropriate subject of knowledge work and who should set research agendas.

These issues relate to other limitations for participatory engagements, especially co-production, within academic research. While there are exceptions (including the above types of PAR research, but see also e.g. Collins *et al*., 2020), decision-making about research questions and design, for example, still tends, to remain in researchers’ hands. Publics or patients are usually involved with more limited questions like design of patient information sheets, and typically only on a study-by-study basis, constraining temporally sustained engagements with research more broadly conceived ( Paylor & McKevitt, 2019). Despite the increasing availability of guidance documents on how to conduct engagements, engagement mandates have not led to the formalisation of training for researchers and healthcare professionals in the skills to carry out ‘good’ engagements. This has facilitated the commercialisation and professionalisation of engagements where ‘independent’ engagement practitioners and organisations are sometimes employed to undertake engagements on researchers’ behalf (see Bherer *et al*., 2017; Pallett, 2019). Yet, professional roles to carry out engagements, within both universities and independent professional engagement agencies, may not have clear career development pathways. A more processual and experimental approach to engagement requires, paradoxically perhaps, the deployment of adequate resources at the infrastructural level (including funding, time, and space) as well as cultural and institutional forms of validation (whether by means of formal accreditation, monetary remuneration, and/or symbolic valorisation of engagement-related labour; see Filipe *et al*., 2017). 

We contend that the feminist tools of reflexivity and positionality can enable us to approach and think differently about the roots, manifestations and mainstreaming of engagements. This includes the contextually conditioned nature of institutionally authorised models of engagements, and the actual motivations and stated justifications around engagements that are being deployed within the confines of institutions. While the mainstreaming of engagements has opened positive, potentially empowering spaces for bringing in new actors and voices into science, institutionalising engagements carries the danger of homogenising and ‘fixing’ engagements into particular, authorised models of ‘good’ or ‘best’ practice. In theorising and enacting engagements, foregrounding positionality and reflexivity enables us to see how mainstreamed institutional models of engagements are embedded in and may reinforce wider power and epistemic structures. This includes interrogation of the relationship between the engager and the engagee, how we conceptualise ‘engaged research,’ and the roles of different actors in the process. We can and should also interrogate the justifications that drive engagements – both our own and more widely – and the implicit as well as explicit motives and interests that engagements may support. We thus call for critical, theoretical and practical reflexivity on the models, motivations, and justifications based on which engagements are undertaken, and for ‘positioning’ engagements as situated activities and processes that are always undertaken from a partial perspective and remain contextual in ways that challenge universalising claims to ‘best practice.’

## Engagements and knowledge production

The institutional context of engagements prompts questions about engagements’ epistemic dimensions: what do engagements, at different moments of the research process, *do* to processes of knowledge production? To what extent are engagements knowledge producing activities, and should they be? What sorts of knowledge might be produced and shared, by whom, for whom, and for what purposes? These questions are centrally connected with the value and positions attributed to different types of knowledge and knowledge producers within engagements and research processes.

Institutional framings and structures around scientific knowledge production tend to delimit what counts as knowledge producing activity, and the types of knowledge that can be generated. Firstly, research governance frameworks often explicitly differentiate between research and engagements, where the former is, for example, subject to ethical oversight while the latter may not be. While being exempt from oversight procedures can be liberating in permitting creativity and experimentation, it can limit what knowledge engagements are able (or allowed) to generate. There are difficulties converting insights from engagements into valid(ated) knowledge, for example, if the engagements have not undergone the institutional ethical review process required for research with human participants, which presents a barrier to publishing findings especially in peer-reviewed journals. Engagements undoubtedly generate knowledge, but when institutional structures are not conducive, it may not be captured in a usable way, limiting the possibilities for learning. How is the boundary between research and engagement drawn, why, and by whom? 

Secondly, institutional framings and structures around scientific knowledge production tend to delimit, explicitly or implicitly, the kind of roles that differently positioned actors can occupy in relation to knowledge; i.e. who ‘counts’ as or is permitted to be a knowledge producer. As noted above, feminist theorists among others have long argued that mainstream scientific epistemologies have privileged the ‘expert’ perspective of trained career scientists – a perspective which has been universal *ised,* presented as objective, and as paradigmatic of (valid) ‘knowledge.’ The effect has been that the knowledges of non-scientists, and especially knowledge arising from subjective experience, have been positioned as not knowledge in the proper sense; ‘improper knowledge.’ Relatedly, various types of engagements often implicitly position certain actors as holders or producers of knowledge, while others are framed as receivers or as sources of data.

In recent decades, however, the epistemic privilege of the conventional ‘expert’ perspective has been challenged including through the emergence of what have been termed ‘professional layperson’ and ‘lay expert’ roles. Those occupying these roles are generally defined in opposition to (conventional) ‘experts’ but are also, by the very nature of their role, taken to possess a particular expertise: positional experience and ability to navigate the engagements environment and effectively provide ‘lay perspectives’ that are expected and looked-for within these contexts ( Kerr *et al*., 2007). A further twist on these roles occurs when academics (e.g. bioethicists or social scientists) or professionals with expertise in healthcare and research are cast as ‘laypersons’ and therefore (expected to) represent ‘lay perspectives’ in relation to health-related knowledge in engagement contexts ( Kerr *et al*., 2007). Similarly, the notion of expertise-by-experience is now commonplace across a range of health governance contexts, but can also generate conflicted, confusing, and sometimes impossible roles for participants ( Meriluoto, 2018). This also raises potentially difficult questions about the extent to which having a particular epistemic standpoint (e.g. patienthood and, as an effect of that position, expertise on illness through experience) means that a person knows ‘more’ or ‘better.’ Inhabiting a particular social location does not necessarily mean that the knowledge arising from that location is more valuable than other, differently located knowledges ( Wylie, 2003), but rather, it reminds of the importance of adopting an epistemological perspective that can engage with a range of epistemic points of view (see also Ackerly & True, 2008).

Such dialogue is especially important when considering challenges around alternative knowledge and epistemic communities, including patient groups, that have the potential to cause harm, such as anti-vaccination groups. Knowledge produced within anti-vaccination groups is often framed as, simply, misinformation and those who promote this information are often framed as, simply, misinformed. Yet, anti-vaccination groups themselves tend to be distrustful of scientific ‘experts’ and their knowledge, and frame their own knowledge as a form of patient empowerment, alternative expertise, and as ‘another way of knowing’ (see e.g. Duchsherer *et al*., 2020; Kata, 2012). Mainstream intervention tactics aiming to address anti-vaccination by countering ‘misinformation’ through scientific ‘facts’ are unlikely to be successful, precisely for the above reasons: these tactics are based on the presumed superiority of scientific knowledge over alterative knowledges, which are in turn framed as not proper knowledge (i.e. as misinformation) – an epistemic power dynamic that anti-vaccination groups generally reject ( Kata, 2012). Thus, effectively engaging with these kinds of potentially harmful alterative knowledges likely requires different tactics that engage with rather than simply discount the epistemic perspectives of those who promote these knowledges. This does not mean embracing potentially harmful knowledges, but rather a dialogical engagement with these knowledges and the epistemic perspectives from which alterative knowledge claims are made. This includes the social and epistemic power relations that may cause anti-vaccination promoters to distrust scientific experts in the first place. 

 Further related questions are raised by the issue of how participants’ ‘lived experience’ should be situated within academic environments ( Banfield *et al*., 2018). Participants’ active roles (e.g. in PAR or co-production research) as more than mere sources of data may demand that they should be directly recognised as knowledge producers, for example through co-authorship of academic papers (see e.g. Bain & Payne, 2015). Yet, incorporating these non-conventional actors into the existing academic hierarchies can be challenging and provoke perhaps uncomfortable questions about the purpose and worth of academic training and credentials. We should also ask whether recognition through modes like co-authorship is meaningful to participants, or whether it is, itself, built on the priorities of academics. Indeed, there may be a need to develop different modes of recognition beyond the academic epistemic and value frameworks. 

 We contend that feminist methodological tools and theoretical approaches can help with the above kinds of challenges. Reflexivity and positioning knowledges, both our own and those of ‘others,’ enables one to think differently about the relationship between engagements and knowledge production, and about the epistemic tensions around different kinds of knowledges and how we can or should engage with (rather than dismiss or work against) these tensions. When one foregrounds the situatedness and contextuality of all knowledge claims and begins from the assumption of knowledge plurality, conceptualising knowledge production in general as requiring dialogical engagement with epistemic plurality becomes imperative. This, in turn, can enable one to interrogate and, potentially, intervene in the epistemic power relations and hierarchies that delimit how engagements are framed in relation to knowledge and how ‘knowledge’ itself is conceptualised and validated. This demands reflexivity from those positioned as ‘experts’ on what shapes their own epistemic starting points and interpretations as well as genuine effort to see the world from another’s point of view ( Haraway, 1988), including from the point of view of those whose knowledges are contradictory or oppositional to one’s own. 

 Applying this to engagements can enable the building of more reciprocal forms of engaging and it entails recognition and interrogation of the fact that there are potentially multiple sets of knowledges at play within any given engagement – not (just) singular ‘lay’ or ‘patient’ and ‘scientist’ but plural knowledges and positions from which knowledge is generated, including those of facilitators, practitioners, funders or commissioners, etc. – and that knowledge progresses dialogically. Conceptualising engagements, through a feminist lens as centrally epistemic practices focuses attention on the epistemic starting points and processes as well as end products of engagements, implying that our valuations of engagement should orient around how different knowledges are generated through engaged research, where, from which positions, and by whom. These issues are also connected with the question of how and why engagements are valued and evaluated.

## Valuing and evaluating engagements

As practices and processes of engagements have been mainstreamed, calls for their evaluation have also grown (see e.g. Oliver *et al*., 2019). Processes of evaluating engagements are often not well documented in existing literature ( Esmail *et al*., 2015). Formal mechanisms for evaluating engagements are, however, increasingly required by funding bodies and institutions, and expected by other stakeholders to develop understanding of individuals’ and groups’ perceptions of engagements, increase awareness, or improve participation rates. The UK Research and Innovation (UKRI) evaluation guide, for example, lists evaluation techniques based on social and market research methods like surveys, interviews, focus groups and discussions with target groups or wider publics ( UKRI, 2011). Yet, the formalisation of evaluating engagements raises further questions around how and why evaluations are undertaken, to whose benefit, whose views are represented and whose may be excluded. 

 Formal evaluation guidelines often define evaluation, implicitly or explicitly, in terms of determining and improving the impact of engagements, where ‘impact’ is often framed in relation to changes or benefits ‘beyond academia,’ as delineated in the REF. The UK MRC (2020), for example, considers that evaluation is done through measuring learning, changes in thinking, and inspiration to know more or get involved by those who are engaged. The information that is collected through evaluation is usually seen as means to increase the value of engagement activities, while ‘value’ is associated with ‘impact.’ Conceptualising engagements in this way as may not, however, fully capture the inherently human aspect of engagements, including relationships, exchange of knowledge and ideas that often results in profound and insightful experiences for those involved, and may not be measurable ( Komporozos-Athanasiou *et al*., 2016). Institutional framings of evaluation can have the effect of not only delimiting what (proper) ‘evaluation’ should look like, but also pre-defining the purpose of evaluation as impact and quality assessment, in ways that can silence the potential of evaluation exercises to produce valuable insight and knowledge, on their own right. Purtell *et al*. (2012) have critiqued the drive to emphasise impact and measurement of engagements without due reflection on the purpose or rationale of engagements in the first place. Boivin *et al*. (2018), moreover, found that publics are often not involved in the development and design of the evaluation tools in the first place.

Feminist approaches to evaluation have applied alternative modes and frameworks of (e)valu(at)ing including multi-vocal and appreciative inquiry methods which aim to elucidate social inequalities and increase the capacity of individuals and groups to effectively represent their views on both engagements and research activities through evaluation. Patton (2002) among others has mapped how feminist evaluation approaches have redefined conventional conceptualisations of evaluation. This includes framing evaluation principally as a means towards greater social justice, using evaluation processes and findings to foster positive change, and challenging conventional criteria for assessing evaluations, including objectivity and the neutrality or independence of the evaluator. Feminist evaluators have also highlighted how different epistemic starting points and rationales for evaluation give rise to different criteria for judging quality and value. Some have stressed how individuals from different social backgrounds approach information and evaluation processes in different ways, showing not just that impact has (or has not) occurred, but also why and how people are affected, how they interpret, perceive, and, indeed, value information in the context of their own lives ( Sielbeck-Bowen *et al*., 2002). Action-based paradigms have sought to mobilise evaluation findings to address structural and procedural processes through which some voices and forms of knowledge are prioritised at the expense of others ( Mertens, 1999). This requires rethinking both evaluation and engagements as forms of action that simultaneously assess, inquire, and aim to act upon inclusion and exclusion processes within research, engagement and evaluation. For example, Beardsley & Hughes Miller (2002) developed a feminist framework to evaluate a women’s substance abuse education programme based on the principle of collaboratively incorporating participants’ voice into the design and implementation of the evaluation, to circumvent epistemic power hierarchies and translate the process and findings into participants’ empowerment. 

The above highlights that evaluation may serve multiple purposes and that the ‘value’ of engagements is less an intrinsic singular property. Instead, it is multi-directional and actively created through engagements and evaluation processes. Beyond the kinds of value gained by institutions – such as potential to improve research quality, symbolic and financial value in showcasing research ‘impact’ beyond academia and increasing success in research funding – value may be gained by participants for example through empowerment or sense of purpose ( Komporozos-Athanasiou *et al*., 2016). These kinds of value may be hard to measure, or immaterial in substance, but nonetheless important to those engaging.

The value gained is also shaped and constrained by the wider social and structural contexts in which it is generated, and in which research and engagements occur. Systemic structural inequalities, including along the lines of gender, race, class, (dis)ability, sexuality, immigration status and other socially significant differences simultaneously embed the institutional contexts in which research and engagements are undertaken, delimit who participates (and does not) in engagements, and whose voices are heard, and views represented in evaluations ( Sielbeck-Bowen *et al*., 2002). For example, people from low-income backgrounds with low educational attainment are significantly less likely to participate in engagements for reasons like lack of resources, confidence, and time, while women are disproportionately disadvantaged by caring responsibilities, and asylum seekers more likely to face language barriers to participation (see What Works Scotland, 2017).

Related critical questions are also raised when the symbolic value gained by institutions is considered in relation to the institutional and social positions of those who undertake the labour of engaging. In addition to public engagement professionals, Boylan *et al*. (2019) have provided insight into the identities of scholars for whom responsibility for engagements is assumed: early-career researchers, postgraduate students and, often, women. They highlighted that the responsibility of engaging tends to be disproportionately borne by junior female staff on fixed-term contracts and qualitative researchers, in ways related to perceptions about the kinds of skills and work that are required for engaging, like empathy and emotional labour. This can include managing the emotional reactions of and care towards those who are engaged with – areas which are socially gendered feminine and, relatedly, perceived as ‘soft skills’ ( Boylan *et al*., 2019). Such disparities in gender and career hierarchy raise additional concerns around how engagements may or may not be valued in ways that reflect wider epistemic and gender hierarchies. These include the mainstream scientific epistemic paradigm that has simultaneously devalued, gendered (as ‘women’s work’), and positioned as epistemically inferior social scientific and humanities approaches to producing knowledge, including the methods primarily used in evaluation of engagements. 

 When we apply the feminist epistemic tools of positionality and reflexivity, evaluation becomes grounded in interrogation of the different kinds of value that engagements can embody and generate for differently positioned subjects. This requires the recognition that one cannot fully measure the value of engagements without reflecting on the processes and justifications of engaging, and the wider social power relations and structural conditions under which they occur, including questions around which voices have been included and excluded in engagements. Engagements serve multiple purposes and can result in multiple kinds of value for different people involved. Taking a feminist perspective encourages one to build on this insight to centre the question of value independent of its capacity to be fixed and measured, to understand and explicate how the value of these processes is plural and situated. Building on feminist epistemological frames and approaches to (e)valu(at)ing enables active concern both for the plural ways in which ‘value’ manifests, and for how the kinds of information are produced through evaluation are epistemically positioned in relation to research and ‘knowledge.’ Variation in how engagements are evaluated is, notably, not intrinsically negative, but is necessary to identify and reflect on tensions and discomforts that arise when different forms of value are prioritised, and to ask what value is obtained, by whom, and what social and structural conditions shape this. 

## Conclusion: towards a feminist philosophy of engagements

This paper has aimed to set out, from our situated perspective, what we see as important outstanding questions and issues around engagements. We have used the notion of ‘engagements’ as a problem concept that can nonetheless capture the plural and overlapping ways in which engagements are enacted, moving beyond attempts to delineate definitions or the scope of what does or should count as engagements to focus on the tensions and possibilities that the multiplicity of engagements evoke.

In considering the roots, current manifestations and mainstreaming of engagements especially within institutional contexts, we argue that there is a need to remain reflexive about how these contexts condition the possibilities for engagements, and we call for critical inquiry into the models, motivations and justifications through which engagements are currently enacted. We are concerned with engagements’ epistemic dimensions, especially questions around how engagements are situated in relation to knowledge production. Thus, we call for ongoing inquiry into the kind(s) of knowledge engagements can or should produce, how this knowledge is or should be captured, who is and is not positioned as a knowledge producer, and what kinds of power relations condition the roles attributed to different subjects. We are also concerned about how engagements are and could be (e)valu(at)ed by different actors, to whose benefit, and how ‘value’ is being conceptualised in these processes. We call for recognition of the diverse kinds of value that engagements can bring to different actors involved, understanding ‘value’ as plural, limiting its capacity to be fixed or measured.

We argue that there is a need for a feminist philosophy of engagements, where ‘philosophy’ entails inquiry into what kind of activity something is and how we should do it, and ‘engagements’ remains problematised but necessarily plural, to leave space open for creative re-thinking of what engagements are or could be. ‘Feminist,’ in turn, entails a philosophy informed by feminist epistemology, including emphasis on social power relations, knowledge production, value generation, and their relationship. We argue that translating existing feminist theoretical and methodological tools into the context of engagements can help us to address the outstanding questions and tensions that we have mapped.

Firstly, feminist notions of positionality can facilitate critical interrogation of knowledge production both in research and engagements, including in relation to (e)valu(at)ing engagements. Starting with the insight that all knowledge arises from a particular perspective shaped by the knowledge producers’ position leads to an understanding of knowledge as situated, plural and partial. This enables us to ask critical questions about who are (not) recognised as knowledge producers, and which knowledges are (not) valid(ated) in research and engagements, including questions about the very distinction between research and engagements as separate(d) activities. This can enable us to recognise, unpack, and name the active roles that many different actors play in the generation of knowledges from research and engagements, and the plural kinds of value attributed to them, and to ask questions about which positionalities and knowledges are not being represented. This can also destabilise conventional epistemic and power differentials between researchers and research participants and expose participants’ knowledge as epistemically salient and valuable.

Secondly, feminist reflexivity can enable us to interrogate critically the epistemic starting points and presumptions that shape how engagements are conceptualised and undertaken, and how these starting points direct the kinds of knowledges that can (not) be produced through them, especially within institutional confines. This can shed critical light on the contextually conditioned nature of institutionally authorised models of engagements, and possible tensions between different motivations and justifications around engagements. Embedding reflexivity into how we conceptualise engagements, and how and why we enact them, can enable us to not only acknowledge how institutional pressures and pre-defined ‘best practice’ models may influence our own practices and motivations for engaging, but it can also enable us to challenge these pressures and models to develop alternative ways of imagining and enacting engagements. Doing so is especially pertinent when understood in the wider context where systemic structural inequalities and hierarches delimit the practical realities of who is included in (and excluded from) engagements. 

A feminist philosophy of engagements advanced along these lines would focus, centrally, on the processes as well as end products of engagements, unpacking how engagements serve multiple purposes, generate plural knowledges, and carry manifold kinds of value that require recognition and interrogation. It would require dialogical engagement with this multiplicity and plurality via interdisciplinary modes of thinking to facilitate the concurrent integration of empirical, epistemic, and normative dimensions in developing accounts of engagements both in theory and praxis. This paper has aimed to carve out the starting points for this work, showing how a feminist philosophy offers avenues for thinking differently about engagements, within and beyond institutionalised models.

## Data availability

No data are associated with this article.
