# Supplementary material for: Towards a feminist philosophy of engagements in health-related research
Source: Wellcome Open Res. 2022 Feb 10;6:58. Originally published 2021 Mar 15. [Version 2] doi: 10.12688/wellcomeopenres.16535.2 (PMC8837807; doi:10.12688/wellcomeopenres.16535.2)
Supplement: Peer review history

# Peer review: Reviewer response for version 2

**DOI:** 10.21956/wellcomeopenres.19447.r48617

**Published:** 2022-02-11

**Authors:** Mary Madden

**Affiliations:** 1 Department of Health Sciences, University of York, York, UK

Congratulations on what is now a focused, reflexive and substantive contribution. I enjoyed reading it and hope it serves as a useful provocation. Note, it was not my expectation that the authors would expand on all the 'threads' in the initial review comments but to point out areas where this reader felt things were left hanging. They have managed this very successfully by tightening the focus and structure of the argument in the revision. The piece also benefits from the inclusion of grounded examples of engagement.

 The ‘paradigm wars’ referred to in the original comments were ongoing crude qualitative/quantitative divisions (discussed in the Oakley work referenced). 

 There are typos in the following sentences if you want to give it a final polish:

- There are not merely labels, but act to shape practices of engaging
- show how and why these tools provide a different les to engagements

Does the article adequately reference differing views and opinions?

Partly

Are all factual statements correct, and are statements and arguments made adequately supported by citations?

Partly

Is the Open Letter written in accessible language?

Yes

Where applicable, are recommendations and next steps explained clearly for others to follow?

Partly

Is the rationale for the Open Letter provided in sufficient detail?

Partly

Reviewer Expertise:

Qualitative health research. PPI. Ex community worker. PhD Women's Studies

I confirm that I have read this submission and believe that I have an appropriate level of expertise to confirm that it is of an acceptable scientific standard.

# Peer review: Reviewer response for version 2

**DOI:** 10.21956/wellcomeopenres.19447.r48616

**Published:** 2022-02-10

**Authors:** Kieran C. O'Doherty

**Affiliations:** 1 Department of Psychology, University of Guelph, Guelph, ON, Canada

I thank the authors for attending to my comments.

Does the article adequately reference differing views and opinions?

Yes

Are all factual statements correct, and are statements and arguments made adequately supported by citations?

Yes

Is the Open Letter written in accessible language?

Yes

Where applicable, are recommendations and next steps explained clearly for others to follow?

Yes

Is the rationale for the Open Letter provided in sufficient detail?

Yes

Reviewer Expertise:

public engagement with science & technology; public deliberation; social psychology; health psychology; bioethics

I confirm that I have read this submission and believe that I have an appropriate level of expertise to confirm that it is of an acceptable scientific standard.

# Peer review: Reviewer response for version 1

**DOI:** 10.21956/wellcomeopenres.18221.r43065

**Published:** 2021-04-28

**Authors:** Kieran C. O'Doherty

**Affiliations:** 1 Department of Psychology, University of Guelph, Guelph, ON, Canada

Thank you for the invitation to review the article, “Towards a feminist philosophy of engagements in health-related research.” The article provides a brief overview and history of the growing emphasis on engagements in health research and argues for grounding ongoing work on engagements on feminist epistemologies and methodologies. The argument for such a feminist philosophy is quite short but, given the authors’ aims “to carve out the starting points for this work,” this does not necessarily detract from the value of the proposal. The argument presented by the authors is compelling and, I believe, an important contribution to scholarship and practice of public engagement. The proposal is also bold and challenges some assumptions that are commonly held as a foundation for the production of scientific knowledge. I feel that these challenges are a welcome, potentially disruptive provocation, and ultimately a constructive contribution.

 I have some minor suggestions for improvements of the manuscript, as well as some thoughts on parts of the argument, which I detail below.

 The authors state that, “The ‘expert’ perspective of trained career scientists has, however, been universalised, presented as objective, and as paradigmatic of (valid) ‘knowledge,’ with the effect that the knowledges of non-scientists, and especially knowledge arising from subjective experience, have been positioned as not knowledge in the proper sense; ‘improper knowledge.’ Relatedly, various types of engagements often implicitly position certain actors as holders or producers of knowledge, while others are framed as receivers or as sources of data.” I agree with the general point. However, I feel that the implied criticism (that the knowledges of non-scientists and that arising from subjective experience should not be regarded as ‘improper knowledge’) needs to be articulated more clearly, and presented with some nuance. For instance, the case that experiential knowledge of illness is a relevant form of knowledge is probably easier to argue than, say, in the case of public engagement on vaccination, whether beliefs about a putative link between autism and the MMR vaccine should be regarded as “proper knowledge.” I don’t have an answer to this question, so I certainly don’t expect the authors of this article to provide one. However, I feel that readers who are not sympathetic to the argument presented by the authors will need a more developed argument to be convinced that there is a problem with the view that scientists are holders of “proper knowledge” and those without such training are only holders of “proper knowledge” insofar as this agrees with accepted scientific knowledge.

 The authors state that, “Further, models of evaluating engagements tend to draw from evidence-based intervention models, where engagements are seen as akin to an intervention to be evaluated.” I believe that this statement requires some qualification and possibly supporting evidence. There are a number of frameworks for evaluation of public engagement that do not conceive of public engagement activity as an intervention. I am thinking here, for example, about the work of Julia Abelson and Ray de Vries and their respective colleagues. It may certainly be that dominant form of evaluations view engagement as an intervention but, if this is so, some qualification would be helpful as to the kind of evaluations the authors have in mind and where they are being deployed.

 Finally, the authors refer to reflexivity several times and reflexivity is also identified as a key principle (or notion) proposed as part of a feminist philosophy of engagements. I feel this is an important point, but its force is somewhat reduced by failing to explain what, precisely, the authors mean by reflexivity. Because there is so much scholarship on reflexivity, the term is associated with different meanings (see for example, Slaney, Tafreshi, & Wu, 2019 ^1^, for an overview of different notions of reflexivity). Clearly explicating what the authors associate with the term would strengthen their point.

Does the article adequately reference differing views and opinions?

Yes

Are all factual statements correct, and are statements and arguments made adequately supported by citations?

Yes

Is the Open Letter written in accessible language?

Yes

Where applicable, are recommendations and next steps explained clearly for others to follow?

Yes

Is the rationale for the Open Letter provided in sufficient detail?

Yes

Reviewer Expertise:

public engagement with science & technology; public deliberation; social psychology; health psychology; bioethics

I confirm that I have read this submission and believe that I have an appropriate level of expertise to confirm that it is of an acceptable scientific standard.

## References

[1] : Philosophical Reflexivity in Psychological Science: Do We Have It? Does It Matter?.2019; 10.1007/978-3-030-25308-0_11 237-256 10.1007/978-3-030-25308-0_11

# Response

**Published:** 2022-01-05

**Authors:** Sonja Erikainen

**Affiliations:** University of Edinburgh, UK

We thank the reviewer for their useful comments and feedback, which we believe have enabled us to improve the paper significantly. We have addressed the comments and feedback as follows:

 Comment: I feel that the implied criticism (that the knowledges of non-scientists and that arising from subjective experience should not be regarded as ‘improper knowledge’) needs to be articulated more clearly, and presented with some nuance. For instance, the case that experiential knowledge of illness is a relevant form of knowledge is probably easier to argue than, say, in the case of public engagement on vaccination, whether beliefs about a putative link between autism and the MMR vaccine should be regarded as “proper knowledge.” […] I feel that readers who are not sympathetic to the argument presented by the authors will need a more developed argument to be convinced that there is a problem with the view that scientists are holders of “proper knowledge” and those without such training are only holders of “proper knowledge” insofar as this agrees with accepted scientific knowledge.

 Response: We have now added a paragraph into the ‘engagements and knowledge production’ section that specifically addresses this issue and outlines out take on it (p. 13-14). 

 Comment: The authors state that, “Further, models of evaluating engagements tend to draw from evidence-based intervention models, where engagements are seen as akin to an intervention to be evaluated.” I believe that this statement requires some qualification and possibly supporting evidence. There are a number of frameworks for evaluation of public engagement that do not conceive of public engagement activity as an intervention. I am thinking here, for example, about the work of Julia Abelson and Ray de Vries and their respective colleagues. It may certainly be that dominant form of evaluations view engagement as an intervention but, if this is so, some qualification would be helpful as to the kind of evaluations the authors have in mind and where they are being deployed.

 Response: We agree that this statement was somewhat too simplistic / reductive, and we have now omitted the statement.

 Comment: Finally, the authors refer to reflexivity several times and reflexivity is also identified as a key principle (or notion) proposed as part of a feminist philosophy of engagements. I feel this is an important point, but its force is somewhat reduced by failing to explain what, precisely, the authors mean by reflexivity. […] Clearly explicating what the authors associate with the term would strengthen their point.

 Response: We have now added a new section on feminist epistemology, where we explicate what we mean by ‘reflexivity’ (and ‘positionality’), and how we apply the notion(s) in this paper (pp. 4-6).

# Peer review: Reviewer response for version 1

**DOI:** 10.21956/wellcomeopenres.18221.r43203

**Published:** 2021-04-08

**Authors:** Mary Madden

**Affiliations:** 1 Department of Health Sciences, University of York, York, UK

Employing ‘engagement’ as an umbrella term for imperatives and activities ‘in the health research environment in the UK and beyond’, the article points to a need for critical interrogation of the concepts and practices around ‘engaging’. The authors call for a philosophy of engagements ‘informed by feminist epistemological and methodological approaches to equitable modes of research participation, knowledge production, and valuing.’ They contend that ‘feminist methodological tools and theoretical approaches’ can help with the kinds of challenges they identify.

 While welcoming more critique in this area and agreeing that feminist epistemology has potentially much to offer in this field, I found little substantive to get hold of here. The authors are arguing for more ‘critical inquiry’ but provide limited in depth critical engagement with the existing philosophy of involvement or engagement, or with the empirical work in the field. The article aspires to ‘move beyond questions of terminology’ to ‘focus on the tensions and possibilities that the multiplicity of engagements evoke’. ‘Engagements’ is said to function as a ‘problem concept’ that ‘can be worked with yet has many uses and no settled meaning…there is analytic value in considering the broad range of engagements together, allowing reflection on the intrinsic tensions, discomforts, and possibilities around doing and researching engagements.’ Maybe, but it also risks compounding vagueness. The article promises a consideration of ‘the roots, present modalities and institutional frameworks that have been erected around engagements,’ but provides a generalised critique/impression of the field not well grounded in specific concepts, practices and places. In order to grasp what was on offer and ‘navigate and make sense of this melee of concepts and practices’, I wanted more clarity on the range of concepts and activities conflated under ‘engagements’. For me, the loss of specificity did not help produce a ‘synthesis that challenges and moves us forward’.

 A call for a philosophy of engagements can imply there are none already there and risks contributing to the constant ‘forgetting’ of knowledge work already undertaken (cf feminist philosopher Mary Daly and current debates about ‘race’).  I wanted deeper, more scholarly engagement with the existing material. There are philosophies of ‘engagement’ in political philosophy (citizen involvement in democratic politics) and a long history of attempts to apply these in the history of participatory approaches to research.  In activism there is much to draw on in the work of environmental movements, ‘development’ politics, community work, informal education, feminist organising, disability politics, anti-racism, ‘patient’ movements- patient and health advocacy and activist movements. Knowledge about feminist and other epistemological and methodological approaches to equitable modes of research participation, knowledge production, and valuing are mentioned here but not actively engaged with. The authors note that ‘engagement activities are being absorbed by large HIC institutions, leaving limited space for alterative conceptualisations and enactments of engagements, including the development of culturally and locally appropriate frame’ without discussing the history of ‘alternative conceptualisation and enactments’ in low and middle income countries. It is also perhaps worth noting the differences in the work arising from STS (often about basic science and technologies linked to health) and from applied health research. Also it is worth considering a more critical approach to the ‘causality’/progress of activisms or scientific breakthroughs in particular locations: ‘This sparked a shift in the relationship between patients and healthcare organisations as well as between research participants and research institutions, as patients and participants increasingly demanded recognition of their agency and rights, including to participate in healthcare and health research decision-making’. Were/are people demanding ‘rights’ or specific services/activities? How/ has this been met - a citizen’s rights agenda or a market/consumerist response?

 Engagement practices are said to be ‘shaped and constrained by the wider social and structural contexts in which they are generated, and in which research and engagements occur’ but there is no sense given of what these actually are or how this works. ‘These structural forces – both of the funding and political landscapes, and the wider societal structures that embed them – are not always stable, creating challenges in obtaining consensus, and leading to changing emphasis and value in engagements around who should be engaged with, why, and what it means to engage. Interesting critical questions are also raised when the symbolic value gained by institutions is considered.’ There is no ‘consensus’ on what ‘engagements’ mean because it covers a set of terms deployed to mean different things. It is not clear why or if ‘consensus’ should be a goal given the messy history and complexity of democratic politics and patient and public involvement in research and the power imbalances (conflicts) therein (see Madden & Speed, 2017 ^1^).

 The institutional angle introduced in the, ‘engagements and knowledge production’ section offers the potential for more focused grounding. Funded health research features the complex, simultaneous involvement of numerous institutions (see e.g. Madden et al 2020 ^2^).  One of the key issues in UK applied health research has been to  distinguish active involvement in the research process from being a participant in a study or taking an interest in/being told about research findings (engagement). A key difference is being able to shape a process/make decisions (power to influence the process) within an increasingly corporatised health research agenda (politicians and corporations are not mentioned as actors in the field but healthcare is a key market and political football). It is not clear to me why disregarding this power distinction is helpful in furthering critique. It might be worth considering how the ‘mainstreaming’ referred to corresponds with institutionalisation. INVOLVE started as a separate campaigning organisation. NIHR Involvement has now taken over from INVOLVE and has re-launched key resources previously produced by INVOLVE. The James Lind Alliance is also now in-house and not campaigning from outside.

 The point about a differentiation between research and engagement made ‘via an equation of research with (valid) knowledge production, and relegation of engagements as not (valid) knowledge producing activities’ needs more clarity. Public engagement with the products of research processes can be exercises in dissemination or ‘impact’ and/or PR/marketing/recruitment exercise for universities. To conduct research on engagement (with the products of research) of the quality of the original research in order to determine the impact or assess the value of such engagement activity is complex and often not in the budget, hence quicker, cheaper market research activities. Points about whether/how (performative) academic metrics like REF in the current mode of production meet people’s real life health concerns in particular contexts are worth pursuing in more depth.

 As it stands the current ‘alternative’ recommended philosophy and its application in ‘engagement’ practices is unclear beyond calls for reflexivity and taking an open mind to evaluation methods (arguably both are already [research] practice norms). It would be useful to clarify whether and how the recommended form of reflexivity differs from that already recommended as standard in qualitative health research texts and evidence that it is absent in current work. The authors ‘contend that feminist methodological tools and theoretical approaches can help with the above kinds of challenges.’ They ‘recommend reflexivity as both a conceptual and practical tool’. It is not clear why this is recommended at the end rather than argued through and applied from the outset.  Doing this would mean applying the emphasis on ‘the situatedness and contextuality of knowledge claims called for’ and more of the reflexivity demanded ‘from those positioned as ‘experts’ on what shapes their own epistemic starting points and interpretations’.

 I wanted to know how/if the feminist ‘epistemic issue of positionality’ referred to here differs from Foucauldian versions. The argument that engagements are required but not valued academic work and carried out by women could be made more clearly. It would be useful to know if there is empirical data available on the growth of PPI/E research labour (as part of research posts) and as a profession (often in marketing departments as part of ‘engagement’)?

  ‘Feminist approaches to evaluation’ are said to ‘have applied alternative modes and frameworks of (e)valu(at)ing… enable[ing] active concern both for the plural ways in which ‘value’ manifests, and for how, why, and what kinds of information are produced through evaluation, as well as how this information is epistemically positioned in relation to research and ‘knowledge.’ Feminist epistemology is another rich field of debate (and major disagreement) and it would be helpful to locate this specifically at this stage of the (gendered) ‘paradigm wars’. Many, including Anne Oakley are concerned with appropriate methods of evaluation rather than ‘alternatives’ per se.  It is a truism (rather than an intrinsically feminist point) ‘that evaluation may serve multiple purposes, including social, cultural, political and financial; and, that the ‘value’ of engagements is less an intrinsic singular property and more something that is multi-directional and actively created through engagements and evaluation processes.’

 Madden, M, Speed, E. Beware Zombies and Unicorns: Toward Critical Patient and Public Involvement in Health Research in a Neoliberal Context.   Frontiers in Sociology 2017 2:7    https:10.3389/fsoc.2017.00007    ISSN=2297-7775 ^1^

 Madden, M, Morris, S, Ogden, M, Lewis, D, Stewart, D, McCambridge, J. Producing co‐production: Reflections on the development of a complex intervention. Health Expect. 2020; 23: 659– 669. https://doi.org/10.1111/hex.13046 ^2^

 Oakley, A (2000) Experiments in Knowing: Gender and Method in the Social Sciences.The New Press ^3^

Does the article adequately reference differing views and opinions?

Partly

Are all factual statements correct, and are statements and arguments made adequately supported by citations?

Partly

Is the Open Letter written in accessible language?

Yes

Where applicable, are recommendations and next steps explained clearly for others to follow?

Partly

Is the rationale for the Open Letter provided in sufficient detail?

Partly

Reviewer Expertise:

Qualitative health research. PPI. Ex community worker. PhD Women's Studies

I confirm that I have read this submission and believe that I have an appropriate level of expertise to state that I do not consider it to be of an acceptable scientific standard, for reasons outlined above.

## References

[1] : Beware Zombies and Unicorns: Toward Critical Patient and Public Involvement in Health Research in a Neoliberal Context. *Frontiers in Sociology* .2017;2: 10.3389/fsoc.2017.00007 10.3389/fsoc.2017.00007

[2] : Producing co-production: Reflections on the development of a complex intervention. *Health Expect* .23(3) : 10.1111/hex.13046 659-669 10.1111/hex.13046 32233053PMC7321726

[3] : Experiments in Knowing: Gender and Method in the Social Sciences. *The New Press* .2000;

# Response

**Published:** 2022-01-05

**Authors:** Sonja Erikainen

**Affiliations:** University of Edinburgh, UK

We thank the reviewer for their useful comments and feedback, which we believe have enabled us to improve the paper significantly. We have addressed the comments and feedback as follows:

 We are very grateful for reviewer one’s extensive engagement with our paper and arguments. Yet, their comments and feedback pull towards many different directions, and we found that addressing them all comprehensively was not possible – the paper would be at risk of losing coherence and direction if we were to pull on all these threads. Their comments and feedback, in many ways because of their wide-ranging nature, have, however, been extremely helpful in enabling us to reflect on and clarify the aims and scope of this paper and better define its parameters and limitations. Thus, a key way in which we have responded to reviewer one is to take a wider lesson across all their comments, and more explicitly and clearly define the scope and terms of our arguments. To do this, we have revised the introduction, added a new section on feminist theory, and revised the other sections in light of this new content. Beyond this, our responses to their comments are as follows:

 Comment: I found little substantive to get hold of here. […] The article […] provides a generalised critique/impression of the field not well grounded in specific concepts, practices and places. In order to grasp what was on offer and ‘navigate and make sense of this melee of concepts and practices’, I wanted more clarity on the range of concepts and activities conflated under ‘engagements’. For me, the loss of specificity did not help produce a ‘synthesis that challenges and moves us forward’.

 Response: We have added some new, more specific examples across the paper, to provide more to ‘get a hold of’ empirically. We have also added a new section (pp. 4-6) to elaborate on our conceptualisation and use of the feminist theoretical concepts we are working with. However, we have retained our use of ‘engagements’ as a problem concept despite the ambiguity and loss of specificity this invites, because one notable point of the paper is to avoid (and, indeed, argue against) fixing or delimiting what ‘engaging’ can (or should) mean, to leave space open for different and alternative ways to think about and enact engagements. Our aim in doing so is precisely to challenge and move beyond terminological delineations and quibbles, and provoke readers to think more expansively (and creatively) about the wide range of things that can be ‘engagements.’ We did, however, omit the notion of ‘synthesis,’ as the reviewer is right in suggesting that what we produce here is, less a synthesis, and more a provocation.

 Comment: A call for a philosophy of engagements can imply there are none already there and risks contributing to the constant ‘forgetting’ of knowledge work already undertaken […] I wanted deeper, more scholarly engagement with the existing material. There are philosophies of ‘engagement’ in political philosophy (citizen involvement in democratic politics) and a long history of attempts to apply these in the history of participatory approaches to research. In activism there is much to draw on in the work of environmental movements, ‘development’ politics, community work, informal education, feminist organising, disability politics, anti-racism, ‘patient’ movements- patient and health advocacy and activist movements.

 Response: We have now added an acknowledgement of the existence of other philosophies of engagement (from political philosophy) (pp. 5-6) but have not engaged with these in any depth, because our focus is on the application of feminist theory to the sphere of engagements.

 We have added more content concerning activist movements and patient mobilisations (pp. 6-7) (also as a way to provide more specific examples into the paper), but not in relation to philosophy of engagements directly as the connection between the kinds of activist movements highlighted by the reviewer and philosophy, as conceptualised in this paper, is only indirect.

 Comment: Knowledge about feminist and other epistemological and methodological approaches to equitable modes of research participation, knowledge production, and valuing are mentioned here but not actively engaged with.

 Response: We have now added a new section on feminist theory into the paper (pp. 4-6), which grounds the paper within feminist epistemology as its theoretical basis, and we have applied this across the paper better.

 Comment: The authors note that ‘engagement activities are being absorbed by large HIC institutions, leaving limited space for alterative conceptualisations and enactments of engagements, including the development of culturally and locally appropriate frame’ without discussing the history of ‘alternative conceptualisation and enactments’ in low and middle income countries. It is also perhaps worth noting the differences in the work arising from STS (often about basic science and technologies linked to health) and from applied health research. Also it is worth considering a more critical approach to the ‘causality’/progress of activisms or scientific breakthroughs in particular locations. Were/are people demanding ‘rights’ or specific services/activities? How/ has this been met - a citizen’s rights agenda or a market/consumerist response?

 Response: We have now revised the relevant section about alternative conceptualisations, including by adding new content on engagements in LMICs and clarifying our arguments related to this (p. 10) (and as a way to provide more specific examples into the paper).

 We have not added content on the difference between STS and applied health research, as this is beyond the scope of the paper and does not directly contribute towards its aims.

 We expanded the section on the legacy of activism to better highlight the wider scope of relevant activism (pp. 6-7) (and as a way to address the above comment concerning activist movements and patient mobilisations).

 Comment: Engagement practices are said to be ‘shaped and constrained by the wider social and structural contexts in which they are generated, and in which research and engagements occur’ but there is no sense given of what these actually are or how this works.

 Response: There was already a statement of what the relevant social structures are, but this has been slightly expanded by providing some examples of how social and structural conditions place barriers to participation (p. 17). We have also expanded the related argument about gender and career hierarches around who undertakes the labour of engaging and why (p. 17).

 Comment: There is no ‘consensus’ on what ‘engagements’ mean because it covers a set of terms deployed to mean different things. It is not clear why or if ‘consensus’ should be a goal given the messy history and complexity of democratic politics and patient and public involvement in research and the power imbalances (conflicts) therein

 Response: We fully agree and did not intend to argue that consensus is or should be to goal – merely that it is difficult to reach. We have, however, now deleted the relevant statement to avoid any misunderstanding in this regard.

 Comment: One of the key issues in UK applied health research has been to distinguish active involvement in the research process from being a participant in a study or taking an interest in/being told about research findings (engagement). A key difference is being able to shape a process/make decisions (power to influence the process) within an increasingly corporatised health research agenda (politicians and corporations are not mentioned as actors in the field but healthcare is a key market and political football). It is not clear to me why disregarding this power distinction is helpful in furthering critique. It might be worth considering how the ‘mainstreaming’ referred to corresponds with institutionalisation.

 Response: We already devote significant space across the paper to interrogating and challenging the distinction between research and engagements, including the many roles that participants can occupy in both (from being a research participant in the conventional sense to being an ‘engagee’ or participating in engagement activities to being a co-producer of knowledge able to make decisions and set agendas). We do not feel that more content on this would add to the paper – indeed, distinctions and power relations embedded in the roles that participants are (and are not) allowed to occupy in the processes of research and engagements are not disregarded in this paper – they are one of our areas of focus.

 While the politics and corporatisation / marketisation of health research are not our focus, we have added an acknowledgement of some of the ways in which corporatisation / marketisation shapes the health research landscape and agendas, through an example of ‘expanded access’ (p. 7). This example is specifically about the question of patients’ and patient advocacy groups’ power to make decisions and influence research processes.

 We have added the word ‘consequently’ in the statement, “in universities, developments like the above constitute the background against which engagements have become increasingly mainstreamed and, consequently, institutionalised,” to highlight that institutionalisation of engagements in universities followed from the developments that have made engagements more mainstream (p. 8). 

 Comment: The point about a differentiation between research and engagement made ‘via an equation of research with (valid) knowledge production, and relegation of engagements as not (valid) knowledge producing activities’ needs more clarity.

 Response: We have revised this sentence to make it clearer (p. 12)

 Comment: Points about whether/how (performative) academic metrics like REF in the current mode of production meet people’s real life health concerns in particular contexts are worth pursuing in more depth.

 Response: This is beyond the scope of this paper, and thus we have not pursued this in more depth.

 Comment: As it stands the current ‘alternative’ recommended philosophy and its application in ‘engagement’ practices is unclear beyond calls for reflexivity and taking an open mind to evaluation methods (arguably both are already [research] practice norms). It would be useful to clarify whether and how the recommended form of reflexivity differs from that already recommended as standard in qualitative health research texts and evidence that it is absent in current work. […] It is not clear why [reflexivity] is recommended at the end rather than argued through and applied from the outset. Doing this would mean applying the emphasis on ‘the situatedness and contextuality of knowledge claims called for’ and more of the reflexivity demanded ‘from those positioned as ‘experts’ on what shapes their own epistemic starting points and interpretations’.

 Response: We have added a new section on feminist theory into the paper (pp. 4-6), which grounds the paper within feminist epistemology as its theoretical basis. This includes elaboration and clarification on the notion of ‘reflexivity’ (and ‘positionality’) that we advocate for, and how they also shape and condition our own knowledge claims. We have then applied this theoretical basis across the paper better (including arguing for and showing the applicability of both reflexivity and positionality throughout the paper), to more directly show how a feminist philosophy offers a different basis and angle for thinking about the kinds of challenges and issues that, we argue, characterise the landscape of engagements.

 Comment: I wanted to know how/if the feminist ‘epistemic issue of positionality’ referred to here differs from Foucauldian versions.

 Response: It has now been clarified that feminist notions of positionality – including ours – often tend to be built on Haraway’s version (p. 5). Elaboration of Foucauldian theory is beyond the scope of this paper.

 Comment: The argument that engagements are required but not valued academic work and carried out by women could be made more clearly. It would be useful to know if there is empirical data available on the growth of PPI/E research labour (as part of research posts) and as a profession (often in marketing departments as part of ‘engagement’)?

 Response: We have added further detail on the gendered labour of engagements and the related power relations around how this labour is (not) valued (p. 17). 

 Empirical data / detail on the growth of PPI/E research labour and profession is outside the scope of this paper beyond the content we already provide on this.

 Comment: Feminist epistemology is another rich field of debate (and major disagreement) and it would be helpful to locate this specifically at this stage of the (gendered) ‘paradigm wars’

 Response: We have now added a new section on feminist epistemology and more directly shown how our arguments are located within and build on feminist epistemology (and which feminist tools we build on, more specifically) (pp. 4-6). We are, however, not sure to which particular ‘paradigm wars’ the reviewer here refers and thus unable to locate our arguments in this regard (for there have been and continue to be many paradigm wars within and against different feminisms).
